# Acupuncture for the prevention of chemotherapy‐induced nausea and vomiting in cancer patients: A systematic review and meta‐analysis

**DOI:** 10.1002/cam4.5962

**Published:** 2023-05-24

**Authors:** Yuqian Yan, Jesús López‐Alcalde, Linxin Zhang, Alexander R. Siebenhüner, Claudia M. Witt, Jürgen Barth

**Affiliations:** ^1^ Institute for Complementary and Integrative Medicine University Hospital Zurich and University of Zurich Zurich Switzerland; ^2^ Faculty of Health Sciences Universidad Francisco de Vitoria (UFV) Madrid Spain; ^3^ Instituto Ramón y Cajal de Investigación Sanitaria (IRYCIS), Unidad de bioestadística clínica, Hospital Universitario Ramón y Cajal, (CIBERESP) Madrid Spain; ^4^ Department for Hematology and Oncology Hirslanden Zurich AG Zurich Switzerland; ^5^ Charité – Universitätsmedizin Berlin, corporate member of Freie Universität Berlin, Humboldt‐Universität zu Berlin, and Berlin Institute of Health, Institute of Social Medicine, Epidemiology and Health Economics Berlin Germany; ^6^ Center for Integrative Medicine University of Maryland School of Medicine Baltimore Maryland USA

**Keywords:** chemotherapy, clinical cancer research, meta‐analysis, side effects

## Abstract

**Purpose:**

To assess the effectiveness and safety of acupuncture for the prevention of chemotherapy‐induced nausea and vomiting (CINV), with a specific intention on exploring sources of between‐study variation in treatment effects.

**Methods:**

MEDLINE, EMBASE, Cochrane CENTRAL, CINAHL, Chinese Biomedical Literature Database, VIP Chinese Science and Technology Periodicals Database, China National Knowledge Infrastructure, and Wanfang were searched to identify randomized controlled trials (RCTs) that compared acupuncture to sham acupuncture or usual care (UC). The main outcome is complete control (no vomiting episodes and/or no more than mild nausea) of CINV. GRADE approach was used to rate the certainty of evidence.

**Results:**

Thirty‐eight RCTs with a total of 2503 patients were evaluated. Acupuncture in addition to UC may increase the complete control of acute vomiting (RR, 1.13; 95% CI, 1.02 to 1.25; 10 studies) and delayed vomiting (RR, 1.47; 95% CI, 1.07 to 2.00; 10 studies) when compared with UC only. No effects were found for all other review outcomes. The certainty of evidence was generally low or very low. None of the predefined moderators changed the overall findings, but in an exploratory moderator analysis we found that an adequate reporting of planned rescue antiemetics might decrease the effect size of complete control of acute vomiting (*p* = 0.035).

**Conclusion:**

Acupuncture in addition to usual care may increase the complete control of chemotherapy‐induced acute vomiting and delayed vomiting but the certainty of evidence was very low. Well‐designed RCTs with larger sample sizes, standardized treatment regimens, and core outcome measures are needed.

## Graphical abstract

Acupuncture in addition to usual care may increase the complete control of chemotherapy‐induced acute vomiting and delayed vomiting but the certainty of evidence was very low. A total of 24 different acupoints had been used, most often ST36, PC6, and CV12.

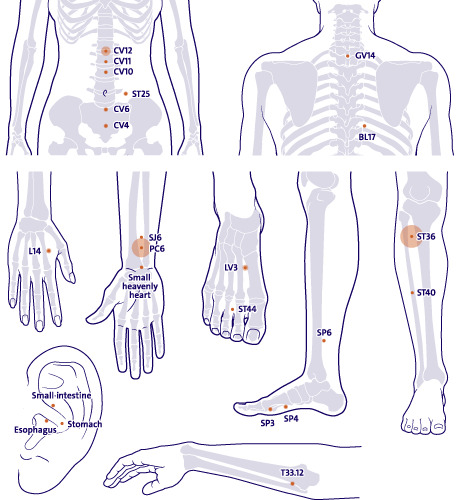

## 1 INTRODUCTION

Chemotherapy‐induced nausea and vomiting (CINV) is one of the most distressing adverse effects among patients undergoing chemotherapy.^1^, ^2^ CINV potentially affects 60% to 80% of patients when left untreated^3^ and the suffering of CINV partly depends on the emetogenicity of chemotherapy agents.^4^ CINV impacts patient's quality of life^5^, ^6^ and may provoke low adherence with chemotherapy regimens that would in consequence compromise treatment efficacy.^6^, ^7^ Prevention of CINV is key as it can reduce morbidity (e.g., anticipatory, refractory, and breakthrough CINV) and healthcare cost.^8^ Numerous prophylactic antiemetics for CINV have been developed and have dramatically improved the prevention of CINV.^7^ The commonly used medications are 5‐hydroxytryptamine3 (5‐HT3) receptorantagonists, neurokinin 1 (NK‐1) receptor antagonists, corticosteroids, and the antipsychotic drug olanzapine.^6^, ^9^ However, the management of CINV remains suboptimal for many patients since about one third of the patients still suffer from CINV under antiemetic drugs.^5^, ^10^, ^11^ There is also a substantial financial costs of antiemetic drugs for the management of CINV.^12^, ^13^, ^14^ To this end, it is helpful to have a multidisciplinary approach to optimize the prevention of CINV.

Acupuncture is a relatively safe medical procedure commonly used to manage cancer‐related side effects for which conventional treatment options are limited.^15^, ^16^, ^17^ While the American Society of Clinical Oncology (ASCO) gives no clear guidance about the use of acupuncture,^9^ the National Comprehensive Cancer Network (NCCN), the German Guideline Program in Oncology, and the Society for Integrative Oncology (SIO) suggested the use acupuncture for CINV.^18^, ^19^, ^20^ An early systematic review published in 2005 assessed the effectiveness of acupuncture‐point stimulation for CINV.^21^ Although the results of this review should be interpreted with caution as this review requires updating, it was pointed out that electroacupuncture may be beneficial in reducing acute vomiting. The beneficial effect of acupuncture was indicated in 2013 by another systematic review.^22^ However, most of the studies in these reviews used outdated antiemetic agents, so their results are not applicable to current practice.

Studies have shown that the likelihood of having CINV depends not only on the intrinsic anti‐cancer treatment properties (e.g., emetogenicity of chemotherapy agents), but relies also on patient factors (e.g., patient characteristics such as female gender and medical conditions such as previous CINV).^4^, ^23^, ^24^ It is critical to investigate the interaction of these moderators alongside acupuncture therapy to explore the generalizability of findings and potential target groups.

The objective of this systematic review and meta‐analysis was to assess the effectiveness and safety of acupuncture in cancer patients schedule to receive chemotherapy for the prevention of CINV, with a specific intention on exploring sources of between‐study variation in treatment effects.

## 2 METHODS

This systematic review followed the Preferred Reporting Items for Systematic Reviews and Meta‐Analyses 2020 statement (checklist see Appendix S2).^25^ The study protocol was prospectively registered on the Open Science Framework^26^ and is available at https://osf.io/ahcwk.

### 2.1 Literature search

We searched the following electronic databases from their inception to June 2020 without language restrictions: MEDLINE, EMBASE, Cochrane CENTRAL, CINAHL, Chinese Biomedical Literature Database, VIP Chinese Science and Technology Periodicals Database, China National Knowledge Infrastructure, and Wanfang. We also screened the reference list of related systematic reviews. Furthermore, we searched the ongoing trials from WHO International Clinical Trials Registry Platform and Chinese Clinical Trial Registry, as well as the conference proceedings from Complementary and Alternative Medicine field from 2015 to March 2021. Search strategy is available in Appendix S3.

### 2.2 Eligibility criteria

We included randomized controlled trials (RCTs) of needle acupuncture compared with sham acupuncture or usual care. Co‐interventions (e.g., antiemetic therapy) were allowed if they were similar in both study arms. Patients should be adults, diagnosed with cancer of any type or stage, scheduled to receive chemotherapy, and not presenting nausea and vomiting before the acupuncture intervention.

### 2.3 Outcome measures

The review outcomes are complete control (no vomiting episodes and/or no more than mild nausea) of nausea and/or vomiting in the acute phase (0 to 24 h), delayed phase (24 to 120 h), and overall phase (within 120 h). We used the maximum follow‐up available for safety assessment and extracted adverse events of acupuncture as reported by study author.

### 2.4 Study screening, data extraction, and risk of bias assessment

References were screened independently by two authors (YY and LZ). After title and abstract screening, full texts were retrieved for the potentially eligible records. Two authors (YY and LZ) independently reviewed the full texts against the inclusion criteria. Two reviewers (YY and LZ) independently extracted data using a standardized online form via Systematic Review Data Repository (https://srdrplus.ahrq.gov) after initially piloted in six studies. Two reviewers (YY, LZ, or JLA) independently assessed the risk of bias with the Cochrane Risk of Bias Tool.^27^ We contacted the study authors via email for additional data. Discrepancies during the screening, data extraction, and risk of bias assessment were solved by consensus between two authors. A third author (JLA) intervened in the case of unresolved disagreements.

### 2.5 Assessment of emetogenicity of chemotherapy treatment

Two authors (YY and AS) independently assessed the potential emetogenicity of chemotherapy regimen in four levels: high emetic risk (>90%), moderate emetic risk (>30% to 90%), low emetic risk (10% to 30%), and minimal emetic risk (<10%). According to the emetic risk table of antineoplastic agents by ASCO guideline,^9^ we first identified the most emetogenic agent in the combination if only one chemotherapy regimen was applied. In studies with multiple chemotherapy regiments, we identified the most emetogenic agent in the combination and then considered the percentage of patients administered with different chemotherapy regimens. We applied 50% as cutoff to determine the overall emetogenicity on the study level. Discrepancies during the assessment of emetogenicity were solved by consensus between two authors.

### 2.6 Data analysis and synthesis of results

We preferably used data from the first chemotherapy cycle for meta‐analysis. We present effects of acupuncture as relative risks (RRs): A RR greater than 1.0 indicates a benefit for complete control of CINV in the acupuncture group. We preferably used the available‐case analysis based on the intention‐to‐treat population.^28^, ^29^, ^30^ We considered statistical pooling when there was homogeneity of comparison group, variable outcome, and predefined CINV phase. For pooling of studies, we used the random‐effects model with Knapp–Hartung adjustment.^31^, ^32^, ^33^ A *p* value <0.05 was considered statistically significant. We assessed statistical heterogeneity with Cochran's *Q* test and measured its magnitude with Higgin's and Thompson's *I* ^2^ statistics where *I* ^2^ ≥ 50% indicated substantial heterogeneity.^34^ We investigated patterns of heterogeneity in the pooled estimates via moderator analysis with mixed‐effects analysis (at least two studies were included in each subgroup). A list of categorical moderators was defined a priori in the study protocol. We added two variables (i.e., rescue medication, adequate training of the intervention provider) based on their clinical relevance. Potential publication bias was assessed in meta‐analyses including at least 10 studies by visual inspection of the funnel plot and the Egger's regression test.^35^, ^36^ One author (AS) made the judgment about outdated and state‐of‐the‐art antiemetics. We used these data in sensitivity analysis by removing studies having administered outdated antiemetics to examine whether statistical significance and pooled effect size changed. All statistical analyses were conducted using R (R Foundation for Statistical Computing, Vienna, Austria, version 4.0.4).^37^ Statistical methods using R packages are detailed in Appendix S4. A replication of the main analyses was done with Stata 17.0 software.^38^

### 2.7 Grading of evidence

We used the Grading of Recommendations, Assessment, Development and Evaluations (GRADE) approach to rate the certainty of evidence.^39^ According to GRADE guidelines, the quality of the evidence starts at high certainty and downgraded by different levels according to risk of bias, consistency, indirectness, and imprecision.^40^ The rating of evidence was done and negotiated by two authors (YY and JLA). We used GRADEpro GDT^41^ to prepare the Summary of Findings (SoF) tables.^42^

## 3 RESULTS

The searches identified 8204 unique citations, among which 268 were assessed potentially eligible at title and abstract screening. Further screening of full texts excluded 220 studies and identified four ongoing studies.^43^, ^44^, ^45^, ^46^ The exclusion reasons of the excluded reports during full‐text screening are listed in Appendix S5. We finally included 38 studies (44 reports) with a total of 2503 patients.^47^, ^48^, ^49^, ^50^, ^51^, ^52^, ^53^, ^54^, ^55^, ^56^, ^57^, ^58^, ^59^, ^60^, ^61^, ^62^, ^63^, ^64^, ^65^, ^66^, ^67^, ^68^, ^69^, ^70^, ^71^, ^72^, ^73^, ^74^, ^75^, ^76^, ^77^, ^78^, ^79^, ^80^, ^81^, ^82^, ^83^, ^84^ The PRISMA flowchart throughout the review process is in Appendix S1.

### 3.1 Characteristics of included studies

**FIGURE 1:**
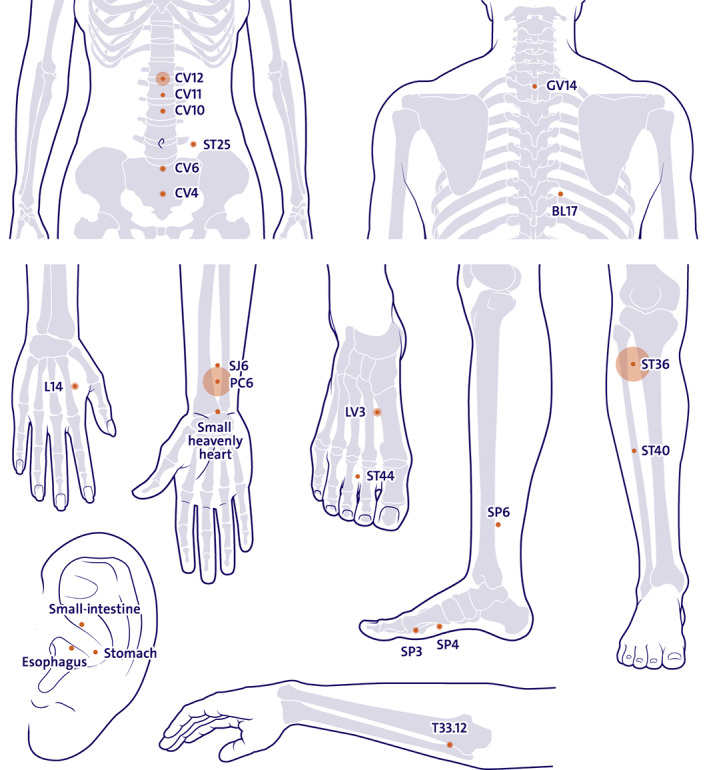
Visualized location of acupoints for chemotherapy‐induced nausea and vomiting (the bubble size represents the frequency of the acupoints being used).

Table 1 summarizes the overall characteristics of the included studies. The descriptive summary of included studies is detailed in Appendix S6. Most studies (84%, *n* = 32)^53^, ^54^, ^55^, ^56^, ^57^, ^58^, ^59^, ^60^, ^61^, ^62^, ^63^, ^64^, ^65^, ^66^, ^67^, ^68^, ^69^, ^70^, ^71^, ^72^, ^73^, ^74^, ^75^, ^76^, ^77^, ^78^, ^79^, ^80^, ^81^, ^82^, ^83^, ^84^ were conducted in China. The remaining studies were conducted in Australia (*n* = 2),^48^, ^51^ Germany (*n* = 2),^49^, ^52^ the United Kingdom (*n* = 1),^47^ and the United States (*n* = 1).^50^ Patient's age ranged from 20 to 82 (based on the 21 studies that informed age). The proportion of male patients ranged from 0% to 80%. Six studies (16%) included only female patients.^48^, ^50^, ^51^, ^52^, ^65^, ^77^ Five studies (13%) included patients with no prior chemotherapy experience.^48^, ^51^, ^52^, ^65^, ^67^ Ten studies (26%) included patients previously had chemotherapy.^47^, ^49^, ^50^, ^53^, ^54^, ^59^, ^63^, ^70^, ^74^, ^77^ More than half of the studies (53%) did not report whether patients received chemotherapy before the enrollment. The first session of acupuncture was administered prior to chemotherapy in 45% of the studies (*n* = 17),^48^, ^49^, ^50^, ^51^, ^53^, ^55^, ^61^, ^64^, ^65^, ^66^, ^68^, ^69^, ^70^, ^75^, ^80^, ^83^, ^84^ and half of the studies (*n* = 19) did not report the initial administered time point of acupuncture.^47^, ^54^, ^56^, ^57^, ^58^, ^59^, ^60^, ^62^, ^63^, ^67^, ^71^, ^72^, ^73^, ^74^, ^76^, ^78^, ^79^, ^81^, ^82^ A so called De‐qi^85^ response was sought by majority of studies (71%, *n* = 27).^48^, ^49^, ^50^, ^51^, ^53^, ^54^, ^55^, ^56^, ^58^, ^59^, ^61^, ^63^, ^64^, ^68^, ^71^, ^72^, ^73^, ^74^, ^75^, ^76^, ^77^, ^78^, ^79^, ^80^, ^81^, ^82^, ^83^ A total of 24 different acupoints were used for CINV; the location and names of these acupoints are visualized on a human body in Figure 1. The bubble size around the point represents the frequency of the acupoints being used. ST36, PC6, and CV12 were the most popular selected acupoints. A sham acupuncture control was attempted in four out of 38 studies, among which three studies were judged likely to blind the patients.^48^, ^49^, ^51^ CINV measurement tools were inconsistently reported. Twenty studies (53%) informed dichotomous outcome data (i.e., number of patients without nausea and / or vomiting);^49^, ^51^, ^53^, ^57^, ^58^, ^59^, ^61^, ^62^, ^65^, ^69^, ^71^, ^72^, ^73^, ^75^, ^76^, ^77^, ^78^, ^79^, ^83^, ^84^ six studies (16%) reported continuous outcome data (i.e., mean/median score of nausea and/or vomiting);^48^, ^51^, ^52^, ^56^, ^59^, ^66^ six studies (16%) reported discrete outcome data (i.e., mean/median episodes of nausea and/or vomiting per person).^48^, ^49^, ^50^, ^51^, ^59^, ^66^ Due to the heterogeneous reporting of continuous and discrete data, we frequently could not pool the data. Therefore, we could meta‐analyze only dichotomous data from 14 studies (37%).^49^, ^51^, ^57^, ^58^, ^59^, ^61^, ^62^, ^65^, ^69^, ^75^, ^77^, ^78^, ^79^, ^84^ The complete control of vomiting and /or nausea was measured with the WHO side effects rating criteria (29%, *n* = 4),^57^, ^62^, ^77^, ^84^ and the NCI Common Terminology Criteria for Adverse Events (CTCAE) (21%, *n* = 3).^61^, ^69^, ^75^ Half of the meta‐analyzed studies (50%) did not report having used a validated tool for measurement. Most studies (86%) did not describe the outcome assessor or if there was interaction between the patient and the study personnel during the outcome assessment. One study detailed that the outcome assessor was the patient who self‐documented the outcome.^51^ In another study, the patient documented the outcome in a patient diary in collaboration with a physician blinded for the group allocation.^49^

**TABLE 1:** Study characteristics summary (*N* = 38).

Study characteristics	No. of studies (%)
**Types of cancer** ^a^	
Lung cancer	12 (32)
Mixed cancer^b^	11 (29)
Breast cancer	6 (16)
Colon cancer	2 (5)
Respiratory system cancer	1 (3)
Testicular cancer	1 (3)
Unclear	5 (13)
**Emetic risk level of chemotherapy regimen** ^c^	
High	13 (34)
High or moderate^d^	12 (32)
Moderate	7 (18)
Low	1 (3)
Minimal	0
Unclear	5 (13)
**Study comparison**	
Acupuncture and usual care vs. usual care	33 (87)
Acupuncture and usual care vs. sham and usual care	4 (11)
Acupuncture and usual care vs. sham and usual care vs. usual care	1 (3)
**Types of usual care**	
Antiemetic therapy	31 (82)
Pain relief medication	1 (3)
Recombinant human granulocyte colony‐stimulating factor	1 (3)
Unclear	5 (13)
**Needle stimulation**	
Manual acupuncture	23 (61)
Electroacupuncture	10 (26)
Both	5 (13)
**Rescue medication**	
Planned to administer additional antiemetics when necessary	10 (26)
Unclear	28 (74)

^a^ The coding represents the cancer diagnosis of the majority of patients (i.e., more than 50%).

^b^ More than one type of cancer patients were included and no cancer diagnoses has more than 50% of patients.

^c^ The coding represents the emetic risk level of the chemotherapy regimen that was used by the majority of patients (i.e., more than 50%).

^d^ Based on the limited study‐level information (e.g., unclear the amount of patients in different chemotherapy regimens, drug name, dosage), we can only be certain that there were no low or minimal emetic risk chemotherapy regimens involved in these studies.

### 3.2 Risk of bias

The risk of bias assessment for included studies is presented in the Appendix S10. Concerning the risk of selection bias, 23 studies (61%) had an adequate random sequence generation.^48^, ^50^, ^51^, ^52^, ^53^, ^56^, ^57^, ^62^, ^65^, ^66^, ^67^, ^69^, ^70^, ^71^, ^72^, ^73^, ^75^, ^77^, ^78^, ^80^, ^82^, ^83^, ^84^ In the remaining 15 studies (39%), the randomization was unclear: The study claimed to be “randomized,” but the method used to generate the random sequence was not reported. The allocation concealment was adequate in five studies (13%)^49^, ^50^, ^52^, ^68^, ^77^ and unclear in the remaining 25 studies (66%). The risk of performance and detection biases were unclear for all the outcomes in two studies (14%),^49^, ^51^ because although a sham acupuncture control was used to blind the patients, the interaction with the acupuncturist may broke the blinding. There was a high risk of performance and detection biases in the remaining studies (86%). The risk of attrition bias was low in fives studies (36%),^49^, ^51^, ^57^, ^61^, ^75^ and unclear in nine (64%).^58^, ^59^, ^62^, ^65^, ^69^, ^77^, ^78^, ^79^, ^84^ The risk of selective outcome reporting was unclear in most studies because we could not find the trial registration (*n* = 36) or because the study had been registered retrospectively (*n* = 1).^48^ One study had a low risk of selective outcome reporting bias.^52^

### 3.3 Effects for complete control of CINV

The meta‐analysis summary statistics of acupuncture for complete control of CINV are listed in Table 2.

**TABLE 2:** Meta‐analysis of treatment effects (complete control [CC] of nausea and vomiting) of acupuncture versus control groups (summary statistics).

Outcomes	No. of Studies	Heterogeneity				Effect Size and 95% Confidence Intervals			Test of Effect Estimates	
		*Q*	*Q‐df*	*p*	*I* ^*2*^	RR	LL	UL	*t*	*p*
Comparison of ACU and UC verus UC										
CC of acute nausea	4	6.01	3	0.111	50.1	2.20	0.66	7.33	2.09	0.128
CC of acute vomiting	10	8.90	9	0.447	0	1.13	1.02	1.25	2.75	0.022
CC of delayed nausea	2	2.36	1	0.125	57.6	3.75	0.00	71,477.12	1.7	0.338
CC of delayed vomiting	10	18.25	9	0.032	50.7	1.47	1.07	2.00	2.77	0.022
Comparison of ACU and UC versus sham ACU and UC										
CC of acute nausea	2	0.31	1	0.575	0	0.87	0.26	2.90	−1.48	0.379
CC of acute vomiting	3	2.55	2	0.280	21.4	1.05	0.72	1.53	0.53	0.647
CC of delayed nausea	1	—	—	—	—	0.59	0.27	1.26	−1.37	0.169
CC of delayed vomiting	2	0.66	1	0.416	0	1.10	0.28	4.36	0.88	0.539

*Note*: a RR > 1 indicates acupuncture increases the complete control rate, a RR < 1 indicates acupuncture decreases the complete control rate.

Abbreviations: ACU, acupuncture; CC, complete control; LL, lower limit; NA, not applicable; Q, Cochrane Q; RR, risk ratio; UC, usual care; UL, upper limit.

#### 3.3.1 Effects of acupuncture in addition to usual care versus usual care (SoF in Table 3)

**TABLE 3:** GRADE summary of findings table.

*Patient or population*: cancer patients schedule to receive chemotherapy					
*Setting*: any					
*Intervention*: acupuncture and usual care					
*Comparison*: usual care					
Outcomes	№ of participants (studies)	Certainty of the evidence (GRADE)	Relative effect (95% CI)	Anticipated absolute effects	
				Risk with usual care	Risk difference with acupuncture and usual care*
Complete control of acute nausea and vomiting—not reported	—	—	—	—	—
Complete control of acute nausea	180 (4 RCTs)	⨁◯◯◯ Very low^a^, ^b^, ^c^	RR 2.20 (0.66 to 7.33)	Moderate risk	
				50 per 100^d^	60 more per 100 (17 fewer to 317 more)
Complete control of acute vomiting	566 (10 RCTs)	⨁◯◯◯ Very low^a^, ^e^	RR 1.13 (1.02 to 1.25)	Moderate risk	
				50 per 100^d^, ^f^	6 more per 100 (1 more to 13 more)
Complete control of delayed nausea and vomiting—not reported	—	—	—	—	—
Complete control of delayed nausea	100 (2 RCTs)	⨁◯◯◯ Very low^a^, ^c^, ^g^	RR 3.75 (0 to 71,477)	Moderate risk	
				50 per 100^d^	138 more per 100 (50 fewer to 3,573,806 more)
Complete control of delayed vomiting	646 (10 RCTs)	⨁◯◯◯ Very low^a^, ^h^, ^i^	RR 1.47 (1.07 to 2.00)	Moderate risk	
				50 per 100^d^, ^j^	24 more per 100 (4 more to 50 more)
Adverse events related to acupuncture	580 (8 RCTs)	⨁⨁◯◯ Moderate^k^	Two studies informed that there were no adverse events (AEs) related to acupuncture. One study reported 90% of AEs due to acupuncture were hematoma, and 2% were pain. One study reported two out of 30 patients who received acupuncture had hematoma. One study reported mild AEs due to acupuncture (18 cases mild pain, four of moderate pain, one of severe needling pain, five of localized bruising, three of localized skin irritation and 12 of exacerbation of chemotherapy‐induced nausea and vomiting). One study reported two patients had AEs due to electroacupuncture (one event like electrical shock sensation one event with aggravated tingling sensation). One study reported there were four patients experienced needling pain during acupuncture therapy, and another study reported there was one patient with mild dizziness from acupuncture.		

*Note*: *Thresholds for clinically important effects (benefit or worsening) based on the absolute risk difference:* Null effect: 0; Clinically irrelevant effect: lower than 10%; Small effect (clinically relevant): from 10% to <20%; Moderate effect: from 20% to <30%; Large effect: from 30%. *GRADE Working Group grades of evidence*: *High certainty*: We are very confident that the true effect lies close to that of the estimate of the effect. *Moderate certainty*: We are moderately confident in the effect estimate: The true effect is likely to be close to the estimate of the effect, but there is a possibility that it is substantially different. *Low certainty*: Our confidence in the effect estimate is limited: The true effect may be substantially different from the estimate of the effect. *Very low certainty*: We have very little confidence in the effect estimate: The true effect is likely to be substantially different from the estimate of effect.

Abbreviations: CI, confidence interval; RR, risk ratio.

^a^ Downgraded due to high risk of bias by two levels because all the studies were at high risk of performance bias and detection bias.

^b^ Downgraded due to imprecision by one level. The 95% CI of the risk difference for the moderate risk scenario is compatible from a worsening of small magnitude to a benefit of large magnitude, including a null effect. In addition, the observed sample size is lower than the optimal information size (estimated at 825 patients, based on a basal risk of 50% and a relative effect of the intervention of 20%, RR = 1.20).

^c^ As the meta‐analysis included less than 10 studies, we were unable to detect publication bias.

^d^ Based on a network meta‐analysis by Piechotta et al. DOI: 10.1002/14651858.CD012775.pub2.

^e^ Downgraded due to high risk of publication bias by one level: Eggers' test indicates the presence of funnel plot asymmetry (*p* = 0.01).

^f^ The risk difference for high risk scenario (30%) was estimated at 4%, 95% CI [1%, 8%], and for a low risk scenario (80%) was estimated at 10%, 95% CI [2%, 20%].

^g^ Downgraded due to imprecision by two levels. The 95% CI of the risk difference for the moderate risk scenario is compatible from a worsening of large magnitude to a benefit of large magnitude, including null effect. In addition, the observed sample size is lower than the optimal information size (estimated at 815 patients, based on a basal risk of 50% and a relative effect of the intervention of 20%, RR = 1.20).

^h^ Downgraded due to inconsistency by one level (*I* ^2^ = 51%, test of heterogeneity *p* = 0.03).

^i^ Downgraded due to high risk of publication bias by one level: Eggers' test indicates the presence of funnel plot asymmetry (*p* = 0.04).

^j^ The risk difference for high risk scenario (30%) was estimated at 14%, 95% CI [2%, 30%], and for a low risk scenario (80%) was estimated at 38%, 95% CI [6%, 80%].

^k^ Downgraded due to high risk of bias by one level because two studies were at high risk of performance bias and detection bias.

* The  risk difference  (and its 95% confidence interval) is based on the assumed risk in the comparison group and the relative effect of the intervention (and its 95% CI).

**FIGURE 2:**
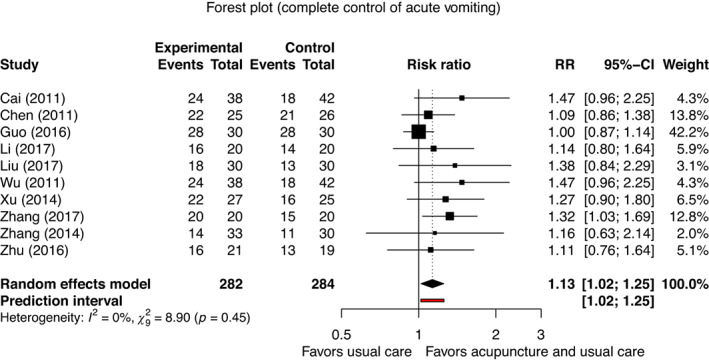
Meta‐analysis for complete control of acute vomiting.

For acute CINV, we found an increased chance of complete control of acute vomiting (RR, 1.13; 95% CI, 1.02 to 1.25; *p* = 0.022; *I* ^*2*^ = 0%, 10 studies, 566 patients; 95% PI, 1.02 to 1.25; forest plot in Figure 2; very low certainty evidence).^57^, ^58^, ^61^, ^62^, ^69^, ^75^, ^77^, ^78^, ^79^, ^84^ Publication bias is suspected (*p* = 0.013; funnel plot of acute vomiting in Appendix S11). However, we did not find an effect for the complete control of acute nausea (RR, 2.20; 95% CI, 0.66 to 7.33; *p* = 0.128; *I* ^*2*^ = 50.1%, four studies, 180 patients; very low certainty evidence).^57^, ^62^, ^75^, ^77^

**FIGURE 3:**
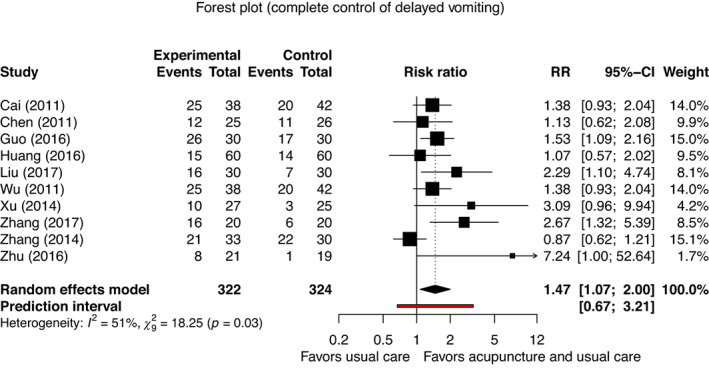
Meta‐analysis for complete control of delayed vomiting.

For delayed CINV, we found an increased chance of complete control of delayed vomiting (RR, 1.47; 95% CI, 1.07 to 2.00; *p* = 0.021; *I* ^*2*^ = 50.7%, 10 studies, 646 patients; 95% PI, 0.67 to 3.21; forest plot in Figure 3; very low certainty evidence).^57^, ^58^, ^61^, ^62^, ^65^, ^69^, ^75^, ^78^, ^79^, ^84^ Publication bias is suspected (*p* = 0.039; funnel plot of delayed vomiting in Appendix S12). However, we did not find an effect for the complete control of delayed nausea (RR, 3.75; 95% CI, 0.00 to 71,477.12; *p* = 0.338; *I* ^*2*^ = 57.6%, two studies, 100 patients; very low certainty evidence).^57^, ^75^

#### 3.3.2 Effects of acupuncture in addition to usual care versus sham acupuncture in addition to usual care (SoF in Appendix S7)

For acute CINV, we did not find an effect for complete control of acute nausea (RR, 0.87; 95% CI, 0.26 to 2.90; *p* = 0.379; *I* ^*2*^ = 0%, two studies, 110 patients; low certainty evidence),^49^, ^51^ and complete control of acute vomiting (RR, 1.05; 95% CI, 0.72 to 1.53; *p* = 0.647; *I* ^*2*^ = 21%, three studies, 182 patients; low certainty evidence).^49^, ^51^, ^59^

For delayed CINV, we did not find an effect for complete control of delayed nausea (RR, 0.59; 95% CI, 0.27 to 1.26; *p* = 0.169; single study, 80 patients; moderate certainty evidence),^49^ and complete control of delayed vomiting (RR, 1.10; 95% CI, 0.28 to 4.36, *p* = 0.539; *I* ^*2*^ = 0%, two studies, 152 patients; very low certainty evidence).^49^, ^59^

No study informed the remaining review outcomes: complete control of chemotherapy‐induced nausea in overall phase; complete control of chemotherapy‐induced vomiting in overall phase; complete control of CINV in acute phase, delayed phase, and overall phase.

### 3.4 Moderator analysis

Moderator subgroups and test results of the complete control of acute vomiting and delayed vomiting are listed in Appendix S8 and S9, respectively. We were unable to explain heterogeneity with our predefined moderators, and we did not find an association between these variables and the treatment effects. However, in an exploratory moderator analysis, we found that an adequate reporting of planned rescue medication might decrease the effect size of complete control of acute vomiting (*p* = 0.035).

### 3.5 Sensitivity analysis

We repeated the analysis by removing studies with outdated antiemetics;^57^, ^69^, ^75^, ^77^ no substantial differences between the primary meta‐analysis were found for acute vomiting (RR, 1.21; 95% CI, 1.05 to 1.40). However, the effect for delayed vomiting was no longer statistically significant (RR, 1.28; 95% CI, 0.85 to 1.91).

We also repeated the analysis by removing studies with low emetic risk of chemotherapy;^79^ no substantial differences between the primary meta‐analysis were found for both acute vomiting (RR, 1.12; 95% CI, 1.01 to 1.24) and delayed vomiting (RR, 1.50; 95% CI, 1.04 to 2.18).

We were unable to undertake more sensitivity analysis as planned in the protocol due to unclear information or lack of studies.

### 3.6 Adverse events related to acupuncture

Twelve out of 38 studies mentioned adverse effects (AEs) of acupuncture, but the reporting of AEs was clear only in 10 (26%). Among these 10 studies, four studies^49^, ^51^, ^57^, ^68^ informed that there were no AEs related to acupuncture. Six studies^48^, ^50^, ^52^, ^53^, ^67^, ^69^ reported AEs of acupuncture with needle pain and localized bruising as predominant AEs.

## 4 DISCUSSION

Our systematic review and meta‐analysis found that acupuncture in addition to usual care, as compared with usual care alone, may increase the chance of complete control of chemotherapy‐induced acute vomiting and delayed vomiting. However, the results did not show effectiveness for chemotherapy‐induced acute nausea and delayed nausea. When acupuncture was compared with sham acupuncture, the results did not show effectiveness for acupuncture in any review outcomes. The certainty of evidence was generally low or very low. No predefined moderators of treatment effects were found.

Our review represents the most comprehensive evidence based on RCTs addressing acupuncture for the prevention of CINV. While a previous systematic review^21^ assessed the effectiveness of acupuncture‐point stimulation on CINV along chemotherapy, such as acupuncture, acupressure, and noninvasive electrostimulation, our review focuses exclusively on needle acupuncture. Moreover, our review focuses on studies for the prevention of CINV by using acupuncture. Despite some slightly different outcome measures, our findings are broadly in line with the conclusion from this previous review. The review authors found that acupuncture is beneficial for chemotherapy‐induced acute vomiting (RR, 0.74; 95% CI, 0.58 to 0.94; *p* = 0.01; four studies); similar to our null finding, they also found no effect for chemotherapy‐induced acute nausea (SMD = 0.02; 95% CI, −0.42 to 0.40; *p* = 0.9; one study). Different treatment effects on nausea and on vomiting are also recognized in conventional usual care. Nausea is a response with dynamic threshold that depends on the interaction of the individual's inherent factors and psychological factors.^86^ Vomiting on the contrary is a yes or no event occurring when stimuli surpass the threshold and it can be easier to control as long as the neuronal signals were reduced to below the threshold.^87^ So nausea is more difficult to manage than vomiting. In addition, researchers less often measure nausea as compared to vomiting,^88^ which was also present in our included studies. One reason for this finding could be that nausea is difficult to measure: First, nausea can only be measured by patients subjectively, which can induce bias when patients were not blinded, or when the blinding was broken due to the interaction with the acupuncturist; second, none of our included studies reported retching independently from nausea or vomiting, and patients may also refer to other gastric symptom as nausea. A careful selection of a user‐friendly nausea‐specific questionnaire could help to manage CINV effectively and allow meaningful assessments in clinical studies.^89^

In our review, a total of 24 different acupoints were used, most of them were known for relieving gastric discomfort. The most frequently used acupoints were ST36, PC6, and CV12. This result is in line with the conclusion from a data mining technology based study^90^ about acupoint selection for CINV. ST36 is a well‐known acupoint for numerous indications, such as enhancing immune system and promoting gastrointestinal functions.^91^, ^92^ PC6 is the most popular anti‐nausea acupoint; it may increase gastric motility and was found to be comparable to antiemetic agents in reducing the incidence of nausea and vomiting.^93^ CV12 is an important acupoint for digestion‐related discomfort.^94^ These three core acupoints for CINV might be a reference for clinical practice. Offering acupuncture might also match with patient preferences, patient expectations towards acupuncture, or good patient‐practitioner relationship.^95^, ^96^ Acupuncture might improve patient outcomes via these contextual factors.^97^ These non‐specific effects may in addition justify the consideration of acupuncture as a treatment option.

Our systematic review shows very uncertain evidence regarding the effectiveness of acupuncture for CINV. Although we included 38 studies, only a small number of data could be meta‐analyzed. Meta‐analyses could be conducted more efficiently if there was an agreed core outcome set on this topic, and research waste could be avoided. In addition, we suggest that primary studies provide information of the effects of acupuncture in specific study populations such as chemo‐naïve, previous poorly controlled CINV during prior chemotherapy, or episodes occurring despite appropriate prophylactic use of antiemetics. This would allow to determine the treatment effects in relevant clinical scenarios. We also want to point out that none of our included studies reported contextual information such as the patient–practitioner relationship and patients' expectations. Trialists could target contextual effects by using a validated expectancy scale,^98^ stratifying the randomization based on prior acupuncture experiences, and recordings of the interaction with the acupuncturist during the intervention. Finally, yet importantly, we speculate the pathophysiology of CINV may vary with different cancer populations and clinical conditions; we therefore highlight the needs to examination moderating effects based on the emetic risk of chemotherapy, state‐of‐the‐art antiemetics, and the application of rescue medication. Even though we were unable to identify any significant effect of the predefined moderators, this does not mean that acupuncture is equally effective between these subgroups. These study‐level data may be potentially important predictors and could be tested with high statistical power in meta‐analysis to inform hypotheses for future primary research.

To our knowledge, this is the first systematic review and meta‐analysis focusing on the preventive effect of acupuncture on CINV. Our exhaustive search for RCTs found 38 included studies involving 2503 patients with 14 studies providing useful data for meta‐analysis, and covered two comparisons with either usual care or sham as control condition. The strengths of our review include explicit eligibility criteria, transparent and comprehensive screening of studies and the extraction of data to increase reproducibility and reliability, the use of GRADE to evaluate the certainty of evidence, and moderator analysis to determine whether the review outcome changes (either in the direction of the effect or in the precision) with respect to predefined explanatory variables.

Despite its strengths, this study has several limitations. First, while delayed CINV is a more common, severe, and hard to manage subtype,^99^ the extraction of delayed data remains challenging. Because there is no consistent reporting of delayed CINV in the included studies, we determined a day within the delayed time period based on the lower incidence of complete control of the intervention group. This approach may underestimate the real complete control of delayed CINV. Second, we collected patient reported adverse events and relied solely on the number of patients without adverse events (i.e., nausea and vomiting) as effect estimate. This assessment may underestimate the subjective experience and the severity of nausea. Finally, we downgraded the certainty of evidence when we observed high risk of bias, imprecision of the effects estimate, inconsistency in study results, and the suspicion of publication bias. On one hand, the methodological challenge lies in the design and the conduct of RCTs in a complex non‐pharmacological intervention like acupuncture,^100^ such as the inability to blind the treatment provider. On the other hand, some analyses included too few studies or patients, especially when acupuncture was compared with sham control, due to which the effect estimates were imprecise. The limitations of these primary studies limited our ability to interpret the data.

In conclusion, our systematic review and meta‐analysis found very low certainty evidence suggesting that acupuncture in addition to usual care, as compared with usual care alone, may increase the chance of complete control of chemotherapy‐induced acute vomiting and delayed vomiting. We did not find an effect when acupuncture was compared with sham acupuncture. To further investigate the prevention of CINV and moderating effects of acupuncture, well‐designed RCTs with large sample sizes, standardized treatment regimens, and core outcome measures are needed.

## AUTHOR CONTRIBUTIONS

**Yuqian Yan:** Conceptualization (equal); data curation (lead); formal analysis (lead); funding acquisition (lead); investigation (equal); methodology (equal); validation (equal); visualization (lead); writing – original draft (lead); writing – review and editing (equal). **Jesús López‐Alcalde:** Conceptualization (equal); formal analysis (equal); investigation (equal); methodology (equal); supervision (supporting); visualization (supporting); writing – original draft (equal); writing – review and editing (equal). **Linxin Zhang:** Conceptualization (supporting); investigation (supporting); writing – original draft (supporting); writing – review and editing (supporting). **Alexander Siebenhüner:** Conceptualization (supporting); investigation (supporting); writing – original draft (supporting); writing – review and editing (supporting). **Claudia M. Witt:** Conceptualization (supporting); investigation (supporting); resources (lead); supervision (supporting); writing – original draft (supporting); writing – review and editing (supporting). **Jürgen Barth:** Conceptualization (equal); formal analysis (equal); investigation (equal); methodology (equal); supervision (lead); validation (equal); writing – original draft (equal); writing – review and editing (equal).

## Supporting information


Appendix S1

Appendix S2

Appendix S3

Appendix S4

Appendix S5

Appendix S6

Appendix S7

Appendix S8

Appendix S9

Appendix S10

Appendix S11

Appendix S12

## Data Availability

Data available in article supplementary material. Data openly available in a public repository that issues datasets with DOIs.
