# Supplementary material for: Acupuncture for the prevention of chemotherapy‐induced nausea and vomiting in cancer patients: A systematic review and meta‐analysis
Source: Cancer Med. 2023 May 24;12(11):12504–17. doi: 10.1002/cam4.5962 (PMC10278514; doi:10.1002/cam4.5962)
Supplement: Supplementary file 1 — Appendix S1 [file CAM4-12-12504-s003.docx]

**Identification of studies via other methods**

**Identification of studies via databases and registers**

Records identified from:

Conferences (n = 3)

Institutions (n = 10)

Citation searching (n = 14)

Records removed *before screening*:

Duplicate records removed (n = 7,838)

Records marked as ineligible by automation tools (n = 0)

Records removed for other reasons (n = 0)

Records identified from:

Databases (n =16,042)

Registers (n = 67)

**Identification**

Records screened

(n = 8,204)

Records excluded (n = 7,936)

Reports not retrieved:

No contact information found (n = 12)

Contacted the author, but no response (n = 1)

Reports not retrieved

(n = 0)

Reports sought for retrieval

(n = 27)

Reports sought for retrieval

(n = 268)

**Screening**

Reports excluded:

Identical with reports from the database retrieval (n = 27)

Reports excluded:

Not eligible study design (n = 21)

Not eligible population (n = 57)

Not eligible intervention (n = 42)

Not eligible comparator (n = 13)

Not eligible study aim (n = 74)

Ongoing studies (n = 4)

Reports assessed for eligibility

(n = 0)

Reports assessed for eligibility

(n = 255)

Studies included in review

(n = 38)

Reports of included studies

(n = 44)

**Included**

*From:*  Page MJ, McKenzie JE, Bossuyt PM, Boutron I, Hoffmann TC, Mulrow CD, et al. The PRISMA 2020 statement: an updated guideline for reporting systematic reviews. BMJ 2021;372:n71. doi: 10.1136/bmj.n71. For more information, visit: <http://www.prisma-statement.org/>
