# Supplementary material for: Acupuncture for the prevention of chemotherapy‐induced nausea and vomiting in cancer patients: A systematic review and meta‐analysis
Source: Cancer Med. 2023 May 24;12(11):12504–17. doi: 10.1002/cam4.5962 (PMC10278514; doi:10.1002/cam4.5962)
Supplement: Supplementary file 3 — Appendix S3 [file CAM4-12-12504-s004.docx]

Appendix 3. Search Strategy

Comprehensive searches were conducted in eight electronic databases:

1) MEDLINE (via PubMed)

2) EMBASE

3) Cochrane CENTRAL

4) CINAHL

5) Chinese Biomedical Literature Database

6) VIP Chinese Science and Technology Periodicals Database

7) China National Knowledge Infrastructure

8) Wanfang Database

The literature search strategy was developed first in PubMed and then translated to the comparable keyword search in other databases. A combination of relevant controlled vocabulary (MeSH - Medical Subject Headings in PubMed) and text / free word was used. All studies from inception until June 2020 were identified. No date or language restrictions were applied.

MEDLINE (via PubMed)

| Search | Query |
| --- | --- |
| #23 | Search: #11 AND #22 |
| #22 | Search: #20 NOT #21 |
| #21 | Search: animals [mh] NOT humans [mh] |
| #20 | Search: #12 OR #13 OR #14 OR #15 OR #16 OR #17 OR #18 OR #19 |
| #19 | Search: groups[Title/Abstract] |
| #18 | Search: trial[Title/Abstract] |
| #17 | Search: randomly[Title/Abstract] |
| #16 | Search: drug therapy[MeSH Subheading] |
| #15 | Search: placebo[Title/Abstract] |
| #14 | Search: randomized[Title/Abstract] |
| #13 | Search: controlled clinical trial[Publication Type] |
| #12 | Search: randomized controlled trial[Publication Type] |
| #11 | Search: #3 AND #10 |
| #10 | Search: #8 OR #9 |

| Search | Query |
| --- | --- |
| #9 | Search: "Neoplasms"[Mesh] |
| #8 | Search: #4 OR #5 OR #6 OR #7 |
| #7 | Search: Mammacarcinom*[Text Word] OR Maltoma*[Text Word] OR Masculinovoblastoma*[Text Word] OR Mastocytoma*[Text Word] OR Medulloblastoma*[Text Word] OR Medullocytoma*[Text Word] OR Medulloepithelioma*[Text Word] OR Medullomyoblastoma*[Text Word] OR Melanoacanthoma*[Text Word] OR Melanoameloblastoma*[Text Word] OR Melanocytoma*[Text Word] OR Melanoma*[Text Word] OR Meningioma*[Text Word] OR Mesenchymoma*[Text Word] OR Mesonephroma*[Text Word] OR Mesothelioma*[Text Word] OR Microcarcinoma*[Text Word] OR Microglioma*[Text Word] OR Micrometastas*[Text Word] OR Myelolipoma*[Text Word] OR [Text Word] OR Myeloma*[Text Word] OR Myoepithelioma*[Text Word] OR Myoblastoma*[Text Word] OR Myoepithelioma*[Text Word] OR Myofibroblastoma*[Text Word] OR Myofibroma*[Text Word] OR Myofibrosarcoma*[Text Word] OR Myolipoma*[Text Word] OR Myoma*[Text Word] OR Myopericytoma*[Text Word] OR Myosarcoma*[Text Word] OR Myxofibroma*[Text Word] OR Myxolipoma*[Text Word] OR Myxoliposarcoma*[Text Word] OR Myxoma*[Text Word] OR Naevus[Text Word] OR Nephroblastoma*[Text Word] OR Nephroma*[Text Word] OR Neurilemmoma*[Text Word] OR Neurilemoma*[Text Word] OR Neuroblastoma*[Text Word] OR Neurocytoma*[Text Word] OR Neuroepithelioma*[Text Word] OR Neurofibroma*[Text Word] OR Neurofibrosarcoma*[Text Word] OR Neurolipocytoma*[Text Word] OR Neuroma*[Text Word] OR Neuronevus[Text Word] OR Neurothekeoma*[Text Word] OR Nevus[Text Word] OR nonhodgkin[Text Word] OR Non-Hodgkin[Text Word] OR Nonseminoma*[Text Word] OR Odontoameloblastoma*[Text Word] OR Odontoma*[Text Word] OR Oligoastrocytoma*[Text Word] OR Oligodendroglioma*[Text Word] OR Oncocytoma*[Text Word] OR Orchioblastoma*[Text Word] OR Osteoblastoma*[Text Word] OR Osteochondroma*[Text Word] OR Osteochondrosarcoma*[Text Word] OR Osteoclastoma*[Text Word] OR Osteofibrosarcoma*[Text Word] OR Osteoma*[Text Word] OR Osteosarcoma*[Text Word] OR Pancreatoblastoma*[Text Word] OR Parachordoma*[Text Word] OR Paraganglioma*[Text Word] OR Paraneoplastic[Text Word] OR Perineurioma*[Text Word] OR Phaeochromocytoma*[Text Word] OR Pheochromoblastoma*[Text Word] OR Pheochromocytoma*[Text Word] OR Pilomatricoma*[Text Word] OR Pilomatrixoma*[Text Word] OR Pinealblastoma*[Text Word] OR Pinealoma*[Text Word] OR Pineoblastoma*[Text Word] OR Pineocytoma*[Text Word] OR Plasmacytom*[Text Word] OR |

| Search | Query |
| --- | --- |
|  | Pneumoblastoma*[Text Word] OR Pneumocytoma*[Text Word] OR Polyembroma*[Text Word] OR Polyhistioma*[Text Word] OR Porocarcinoma*[Text Word] OR Poroma*[Text Word] OR Reninoma*[Text Word] OR Reticuloendothelioma*[Text Word] OR Reticulohistiocytoma*[Text Word] OR Retinoblastoma*[Text Word] OR Rhabdomyoma*[Text Word] OR Rhabdomyosarcoma*[Text Word] OR Rhabdosarcoma*[Text Word] OR Sarcoid*[Text Word] OR Sarcoma*[Text Word] OR Schwannoma*[Text Word] OR Seminoma*[Text Word] OR Somatostaminoma*[Text Word] OR Somatotropinoma*[Text Word] OR Spermatocynoma*[Text Word] OR Spiradenoma*[Text Word] OR Spongioblastoma*[Text Word] OR Steatocystoma*[Text Word] OR Subependymoma*[Text Word] OR Syringadenoma*[Text Word] OR Syringoma*[Text Word] OR Teratocarcinoma*[Text Word] OR Teratoma*[Text Word] OR Thecoma*[Text Word] OR Thymolipoma*[Text Word] OR Thymoma*[Text Word] OR Trichilemmoma*[Text Word] OR Trichoadenoma*[Text Word] OR Trichoblastoma*[Text Word] OR Trichodiscoma*[Text Word] OR Trichoepithelioma*[Text Word] OR Trichofolliculoma*[Text Word] OR Tricholemmoma*[Text Word] OR Vipoma*[Text Word] OR Waldenstrom*[Text Word] OR Xanthoastrocytoma*[Text Word] OR Xanthofibroma*[Text Word] OR Xanthogranuloma*[Text Word] OR Xanthoma*[Text Word] OR Xanthosarcoma*[Text Word] |
| #6 | Search: Dentinoma*[Text Word] OR Dermatofibroma*[Text Word] OR Dermatofibrosarcoma*[Text Word] OR Dictyoma*[Text Word] OR Dysgerminoma*[Text Word] OR Dyskeratoma*[Text Word] OR Dysplasia*[Text Word] OR Dysplastic*[Text Word] OR Ectomesenchymoma*[Text Word] OR Elastofibroma*[Text Word] OR Enchondroma*[Text Word] OR Endothelioma*[Text Word] OR Ependymoblastoma*[Text Word] OR Ependymoma*[Text Word] OR Epitheliom*[Text Word] OR Erythroleukaemia*[Text Word] OR Erythroleukemia*[Text Word] OR Esthesioneuroblastoma*[Text Word] OR Esthesioneuroepithelioma*[Text Word] OR Fibroadenoma*[Text Word] OR Fibroadenosarcoma*[Text Word] OR Fibroelastoma*[Text Word] OR Fibroepithelioma*[Text Word] OR Fibrofolliculoma*[Text Word] OR Fibrolipoma*[Text Word] OR Fibroliposarcoma*[Text Word] OR Fibroma*[Text Word] OR Fibromyxoma*[Text Word] OR Fibroodontoma*[Text Word] OR Fibrosarcoma*[Text Word] OR Fibro- sarcoma*[Text Word] OR Fibrothecoma*[Text Word] OR Fibroxanthoma*[Text Word] OR Fibroxanthosarcoma*[Text Word] OR Ganglioblastoma*[Text Word] OR Gangliocytoma*[Text Word] OR Ganglioglioma*[Text Word] OR Ganglioneuroblastoma*[Text Word] OR Ganglioneurofibroma*[Text Word] OR Ganglioneuroma*[Text Word] OR Gastrinoma*[Text Word] OR Germinoma*[Text Word] OR |

| Search | Query |
| --- | --- |
|  | Glioblastoma*[Text Word] OR Gliofibroma*[Text Word] OR Glioma*[Text Word] OR Glioneuroma*[Text Word] OR Gliosarcoma*[Text Word] OR Glomangioma*[Text Word] OR Glomangiomyoma*[Text Word] OR Glomangiosarcoma*[Text Word] OR Glucagonoma*[Text Word] OR Gonadoblastom*[Text Word] OR Gonocytoma*[Text Word] OR Granuloma*[Text Word] OR Gynandroblastoma*[Text Word] OR Haemangioblastoma*[Text Word] OR Hemangioblastoma*[Text Word] OR Hemangiom*[Text Word] OR Haemangiom*[Text Word] OR Hemangiopericytom*[Text Word] OR Haemangiopericytom*[Text Word] OR Hemangioendothelioma*[Text Word] OR Haemangioendothelioma*[Text Word] OR Hemangioendotheliosarcoma*[Text Word] OR Haemangioendotheliosarcoma*[Text Word] OR Hemangiopericytoma*[Text Word] OR Haemangiopericytoma*[Text Word] OR Hemangiosarcoma*[Text Word] OR Haemangiosarcoma*[Text Word] OR Hepatoblastom*[Text Word] OR Hepatocarcinoma*[Text Word] OR Hepatocholangiocarcinoma*[Text Word] OR Hepatoma*[Text Word] OR Hibernoma*[Text Word] OR Hidradenoma*[Text Word] OR Histiocytoma*[Text Word] OR Hodgkin*[Text Word] OR Hydradenoma*[Text Word] OR Hypernephroma*[Text Word] OR Immunocytoma*[Text Word] OR Keratoacanthoma*[Text Word] OR Leiomyoblastoma*[Text Word] OR Leiomyofibroma*[Text Word] OR Leiomyoma*[Text Word] OR Leiomyosarcoma*[Text Word] OR Leukemia*[Text Word] OR Leukaemia*[Text Word] OR Leukoplakia*[Text Word] OR Lipoadenoma*[Text Word] OR Lipoblastoma*[Text Word] OR Lipoma*[Text Word] OR Liposacoma*[Text Word] OR Lymphom*[Text Word] OR Lymphangioendothelioma*[Text Word] OR Lymphangioma*[Text Word] OR Lymphangiomyoma*[Text Word] OR Lymphangiosarcoma*[Text Word] OR Lymphoepithelioma*[Text Word] OR Lymphoma*[Text Word] OR Lymphoproliferation[Text Word] OR Lymphoscintigraphy[Text Word] |
| #5 | Search: Acanthoma*[Text Word] OR Adamantinoma*[Text Word] OR Adenoacanthoma*[Text Word] OR Adenocarcinoma*[Text Word] OR Adenoma*[Text Word] OR Adenolipoma*[Text Word] OR Adenolymphoma*[Text Word] OR adenomatous[Text Word] OR Adenomyoepithelioma*[Text Word] OR Adenomyoma*[Text Word] OR Adenosarcoma*[Text Word] OR Aesthesioneuroblastoma*[Text Word] OR Ameloblastoma*[Text Word] OR Amyloidosis[Text Word] OR Anaplasia[Text Word] OR Androblastoma*[Text Word] OR Angioblastoma*[Text Word] OR Angioendothelioma*[Text Word] OR Angiofibroma*[Text Word] OR Angiofibrosarcoma*[Text Word] OR Angiokeratoma* Angioleiomyoma*[Text Word] OR Angiolipoma*[Text Word] OR Angioma*[Text Word] OR |

| Search | Query |
| --- | --- |
|  | Angiomyolipoma*[Text Word] OR Angiomyoma*[Text Word] OR Angiomyxoma*[Text Word] OR Angioreticuloma*[Text Word] OR Angiosarcoma*[Text Word] OR Apudoma*[Text Word] OR Argentaffinoma*[Text Word] OR Arrhenoblastoma*[Text Word] OR Astroblastoma*[Text Word] OR Astrocytoma*[Text Word] OR Astroglioma*[Text Word] OR Baltoma*[Text Word] OR Basiloma*[Text Word] OR Carcinosarcoma*[Text Word] OR Cavernoma*[Text Word] OR Cementoma*[Text Word] OR Ceruminoma*[Text Word] OR Chemodectoma*[Text Word] OR Cholangiocarcinoma*[Text Word] OR Cholangiohepatoma*[Text Word] OR Cholangioma*[Text Word] OR Cholangiosarcoma*[Text Word] OR Chondroblastoma*[Text Word] OR Chondro-Blastoma*[Text Word] OR Chondroma*[Text Word] OR Chondrosarcoma*[Text Word] OR Chondro-sarcoma*[Text Word] OR Chordoma[Text Word] OR Chorioadenoma*[Text Word] OR Chorioangioma*[Text Word] OR Choriocarcinoma*[Text Word] OR Chorioepithelioma*[Text Word] OR Choristoma*[Text Word] OR Cementoma*[Text Word] OR Collagenoma*[Text Word] OR Comedocarcinoma*[Text Word] OR Condyloma*[Text Word] OR Corticotropinoma*[Text Word] OR Craniopharyngioma*[Text Word] OR Cylindroma*[Text Word] OR Cyst?[Text Word] OR Cystadenocarcinoma*[Text Word] OR Cystadenofibroma*[Text Word] OR Cystadenoma*[Text Word] OR Cystoma*[Text Word] OR Cystosarcoma*[Text Word] |
| #4 | Search: Cancer*[Text Word] OR Neoplas*[Text Word] OR Carcinom*[Text Word] OR Carcinogen*[Text Word] OR Malignan*[Text Word] OR Tumor*[Text Word] OR tumour*[Text Word] OR Anti-cancer[Text Word] OR Anticancer[Text Word] OR Anti-carcino*[Text Word] OR Anticarcino*[Text Word] OR Antitumor[Text Word] OR Antitumour[Text Word] OR Anti-tumor[Text Word] OR Anti-tumour[Text Word] OR Tumor-inhibit*[Text Word] OR Tumour-inhibit*[Text Word] OR Hyperplas*[Text Word] OR Metastas*[Text Word] OR Metastatic[Text Word] OR Metaplasia[Text Word] OR Oligometasta*[Text Word] OR Oligorecurren*[Text Word] OR Anticarcinogen*[Text Word] OR Antimutagenesis[Text Word] OR Anti-neoplastic[Text Word] OR Antineoplastic[Text Word] OR Antimetastatic[Text Word] OR Anti- metastatic[Text Word] OR Antioncogene[Text Word] OR Metastasectomy[Text Word] OR Oncolog*[Text Word] OR Oncogen*[Text Word] OR Oncolytic[Text Word] OR Lymph node excision*[Text Word] OR Sentinel lymph node*[Text Word] OR mammography[Text Word] OR mastectomy [Text Word] |
| #3 | Search: #1 OR #2 |

| Search | Query |
| --- | --- |
| #2 | Search: (("Acupuncture"[Mesh] OR "Acupuncture Therapy"[Mesh]) OR "Acupressure"[Mesh]) |
| #1 | Search: "acupunct*"[Text Word] OR "electro acupunct*"[Text Word] OR "electroacupunct*"[Text Word] OR "electric stimulation*"[Text Word] OR "acupoint*"[Text Word] OR "acu point*"[Text Word] OR "acustimulation"[Text Word] OR "neiguan"[Text Word] OR "P6"[Text Word] OR "P- 6"[Text Word] OR "wristband"[Text Word] OR "wrist- band"[Text Word] OR "acupotom*"[Text Word] OR "auriculotherap*"[Text Word] OR "meridian*"[Text Word] OR "Ching Lo"[Text Word] OR "Jing Luo"[Text Word] OR "Jingluo"[Text Word] OR "Moxibustion"[Text Word] OR "Moxabustion"[Text Word] OR "pharmacoacupunct*"[Text Word] OR "pharmaco acupunct*"[Text Word] OR "pharmacopunct*"[Text Word] OR "Dequi"[Text Word] OR "acupress*"[Text Word] OR "shiatsu"[Text Word] OR "Tui Na"[Text Word] OR "zhi ya"[Text Word] |
