# Supplementary material for: Acupuncture for the prevention of chemotherapy‐induced nausea and vomiting in cancer patients: A systematic review and meta‐analysis
Source: Cancer Med. 2023 May 24;12(11):12504–17. doi: 10.1002/cam4.5962 (PMC10278514; doi:10.1002/cam4.5962)
Supplement: Supplementary file 4 — Appendix S4 [file CAM4-12-12504-s012.docx]

Appendix 4. Statistical Methods for Meta-Analysis Using R

Package “meta”^1,2^ : Effect sizes were pooled using the metabin function. We anticipated between-study variance / heterogenity in the effect sizes and used the random-effects model. We chose the Paule-Mandel estimator to calculate tau-square,^3^ this method is recommended for binary effect size data.^4^ We applied the Hartung-Knapp adjustment to calculate the confidence interval of the pooled effect estimates,^5,6^ this adjustment is recommended as it is more accurate to reflect the random-effect model especially when the number of studies is small.^7,8^ For statistically significant findings, we presented the prediction interval for estimating the effect that can be expected in future studies.^9,10^ Forest plots were created by using the forest.meta function. Potential publication bias was assessed in meta-analyses including at least ten studies by visual inspection of the funnel plot and the Egger’s regression test.^11,12^ Funnel plots were created by using the funnel.meta function.

Package “metafor”^13^: The moderator analysis was conduced using the rma function. We used the inverse variance method by using the escalc function for calculating the log RR and its corresponding sampling variances, the data was then supplied to the rma function.

Package “robvis”^14^: Risk of bias plot was created by using the rob_traffic_light function with the generic template.

References

1. Balduzzi S, Rücker G, Schwarzer G. How to perform a meta-analysis with R: a practical tutorial. *Evid Based Ment Health*. Nov 2019;22(4):153-160. doi:10.1136/ebmental-2019-300117

2. Harrer M, Cuijpers P, Furukawa TA, Ebert DD. *Doing meta-analysis with R: a hands-on guide*. 1st ed. Chapman & Hall/CRC Press; 2021.

3. Paule RC, Mandel J. Consensus values and weighting factors. *J Res Natl Bur Stand (1977)*. Sep-Oct 1982;87(5):377-385. doi:10.6028/jres.087.022

4. Veroniki AA, Jackson D, Viechtbauer W, et al. Methods to estimate the between-study variance and its uncertainty in meta-analysis. *Res Synth Methods*. Mar 2016;7(1):55-79. doi:10.1002/jrsm.1164

5. Knapp G, Hartung J. Improved tests for a random effects meta-regression with a single covariate. *Stat Med*. Sep 15 2003;22(17):2693-710. doi:10.1002/sim.1482

6. Sidik K, Jonkman JN. A simple confidence interval for meta-analysis. *Stat Med*. Nov 15 2002;21(21):3153-9. doi:10.1002/sim.1262

7. Borenstein M. Section 7.5: Knapp-Hartung adjustment. *Common mistakes in meta-snalysis and how to avoid them*. 2019.

8. Langan D, Higgins JPT, Jackson D, et al. A comparison of heterogeneity variance estimators in simulated random-effects meta-analyses. *Res Synth Methods*. Mar 2019;10(1):83-98. doi:10.1002/jrsm.1316

9. IntHout J, Ioannidis JP, Rovers MM, Goeman JJ. Plea for routinely presenting prediction intervals in meta-analysis. *BMJ Open*. Jul 12 2016;6(7):e010247. doi:10.1136/bmjopen-2015-010247

10. Guddat C, Grouven U, Bender R, Skipka G. A note on the graphical presentation of prediction intervals in random-effects meta-analyses. *Syst Rev*. Jul 28 2012;1:34. doi:10.1186/2046-4053-1-34

11. Sterne JAC, Egger M, Moher D. Chapter 10. Addressing reporting biases. In: Higgins JT, Green S, editor(s). . *Cochrane Handbook of Systematic Reviews of Intervention Version 510 (updated March 2011)*. 2011.

12. Egger M, Davey Smith G, Schneider M, Minder C. Bias in meta-analysis detected by a simple, graphical test. *BMJ*. Sep 13 1997;315(7109):629-34. doi:10.1136/bmj.315.7109.629

13. Viechtbauer W. Conducting Meta-Analyses in R with the metafor Package. *Journal of Statistical Software*. 08/05 2010;36(3):1 - 48. doi:10.18637/jss.v036.i03

14. McGuinness LA. Risk of Bias Plots. In Harrer, M., Cuijpers, P., Furukawa, T.A., & Ebert, D.D., Doing meta-analysis with R: a hands-on guide (online version). <https://bookdown.org/MathiasHarrer/Doing_Meta_Analysis_in_R/rob-plots.html>.
