# Supplementary material for: Acupuncture for the prevention of chemotherapy‐induced nausea and vomiting in cancer patients: A systematic review and meta‐analysis
Source: Cancer Med. 2023 May 24;12(11):12504–17. doi: 10.1002/cam4.5962 (PMC10278514; doi:10.1002/cam4.5962)
Supplement: Supplementary file 5 — Appendix S5 [file CAM4-12-12504-s009.docx]

Appendix 5. Excluded Reports in Full-Text Screening Stage with Exclusion Reasons

| **Study** | **Reason for Exclusion** |
| --- | --- |
| An 2011 | Not eligible comparator: acupuncture + sevoflurane vs. complete anesthesia |
| Anna 2012 | Not eligible population: radiotherapy |
| Annoamous 2019 | Not eligible study design: not randomized controlled trial |
| Asadpour 2017 | Not eligible population: radiotherapy |
| Asadpour 2017 | Not eligible population: radiotherapy (Protocol of an excluded study - Asadpour 2017) |
| Asadpour 2018 | Not eligible population: radiotherapy (Multi report of an excluded study - Asadpour 2017) |
| Bao 2019 | Not eligible study aim: irrelevant study aim, and no nausea/vomiting outcome reported (Multi report of an excluded study - Bao T 2018) |
| Bao 2020 | Not eligible study aim: irrelevant study aim, and no nausea/vomiting outcome reported |
| Bao T 2009 | Not eligible study design: not randomized controlled trial |
| Bao T 2013 | Not eligible study design: not randomized controlled trial |
| Bao T 2018 | Not eligible study aim: irrelevant study aim, and no nausea/vomiting outcome reported |
| Bi YZ 2008 | Not eligible intervention: chinese patent medicine |
| Cai 2013 | Not eligible study aim: irrelevant study aim, and no nausea/vomiting outcome reported |
| Cao 2015 | Not eligible comparator: comparison between acupuncture and electro acupuncture |
| Cao CQ 2007 | Not eligible intervention: acupressure |
| Cao MC 2015 | Not eligible study aim: irrelevant study aim, and no nausea/vomiting outcome reported |
| Capodice 2010 | Not eligible study design: not randomized controlled trial |
| Carlsson 2000 | Not eligible population: no chemotherapy scheduled |
| Chai 2015 | Not eligible comparator: comparison between different expertise of acupuncturepuncturists |
| Chang H 2017 | Not eligible study aim: irrelevant study aim, and no nausea/vomiting outcome reported |
| Chen 2020 | Not eligible intervention: transcutaneous electrical acupuncturepoint stimulation (TEAS) |
| Chen DQ 2015 | Not eligible comparator: acupuncture vs. active control (metoclopramide) |
| Chen HY 2012 | Not eligible study aim: irrelevant study aim, and no nausea/vomiting outcome reported |
| Chen JB 2016 | Not eligible intervention: acupuncture was administered after the chemotherapy, thus it's not likely for the prevention of CINV |
| Chen JJ 2016 | Not eligible intervention: moxibustion |
| Chen PT 2016 | Not eligible population: radiochemotherapy |
| Chen WB 2018 | Not eligible study aim: irrelevant study aim, and no nausea/vomiting outcome reported |
| Chen XQ 2015 | Not eligible study aim: irrelevant study aim, and no nausea/vomiting outcome reported |
| Chen XY 2013 | Not eligible population: radiotherapy |
| Chen XZ 2016 | Not eligible population: all patients were experiencing chemotherapy induced nausea and/or vomiting at baseline (i.e. before the study) |
| Cheng C 2017 | Not eligible population: radiochemotherapy |
| Chokshi 2017 | Not eligible population: children |
| Choo 2006 | Not eligible study design: not randomized controlled trial |
| Cummings 2001 | Not eligible study design: not randomized controlled trial |
| D'Alessandro 2019 | Not eligible study aim: irrelevant study aim, and no nausea/vomiting outcome reported |
| da Silva Varejão 2016 | Not eligible population: children |
| Dan M 2018 | Not eligible intervention: herbal medicine |
| Dang D 2014 | Not eligible study aim: irrelevant study aim, and no nausea/vomiting outcome reported |
| Deng G 2011 | Not eligible population: no chemotherapy scheduled (Multi report of an excluded study - Deng G 2013) |
| Deng G 2013 (1) | Not eligible population: no chemotherapy scheduled |
| Deng G 2013 (2) | Not eligible study aim: irrelevant study aim, and no nausea/vomiting outcome reported |
| Deng G 2017 | Not eligible intervention: acupuncture was administered after the chemotherapy, thus it's not likely for the prevention of CINV (Multi report of an excluded study - Deng G 2018) |
| Deng G 2018 | Not eligible intervention: acupuncture was administered after the chemotherapy, thus it's not likely for the prevention of CINV |
| Deng G 2020 | Not eligible study aim: irrelevant study aim, and no nausea/vomiting outcome reported |
| Deng LY 2016 | Not eligible population: radiotherapy |
| Deng SM 2014 | Not eligible intervention: moxibustion |
| Dilaveri 2020 | Not eligible population: no chemotherapy scheduled |
| Ding L 2011 | Not eligible study design: not randomized controlled trial |
| Ding YH 2015 | Not eligible population: patients age range 17-71, and the data from adult patients were not separately reported |
| Dundee 1988 | Not eligible comparator: acupuncture vs. active control (metoclopramide) |
| Dymackova 2020 | Not eligible population: radiochemotherapy |
| Enblom 2011 (1) | Not eligible population: radiotherapy |
| Enblom 2011 (2) | Not eligible population: radiotherapy |
| Enblom 2012 | Not eligible population: radiotherapy |
| Enblom 2017 | Not eligible population: radiotherapy |
| Fan RZ 2002 | Not eligible intervention: psychological counseling |
| Fitzpatrick 1988 | Not eligible study design: not randomized controlled trial |
| Fu J 2006 | Not eligible intervention: electrical stimulation without needle insertion |
| Fu YH 2014 | Not eligible study aim: irrelevant study aim, and no nausea/vomiting outcome reported |
| Gottschling 2008 | Not eligible population: children |
| Gu 2009 | Not eligible intervention: acupressure |
| Gu 2019 | Not eligible population: no chemotherapy scheduled. Undergoing surgery |
| Guo D 2011 | Not eligible population: patients age range 9-72, and the data from adult patients were not separately reported |
| Guo D 2012 | Not eligible population: patients age range 9-72, and the data from adult patients were not separately reported (Multi report of an excluded study - Guo D 2011) |
| Guo LY 2014 | Not eligible study aim: irrelevant study aim, and no nausea/vomiting outcome reported |
| Han 2010 | Not eligible study aim: irrelevant study aim, and no nausea/vomiting outcome reported |
| Han 2017 | Not eligible study aim: irrelevant study aim, and no nausea/vomiting outcome reported |
| He PS 2017 | Not eligible population: all patients were experiencing chemotherapy induced nausea and/or vomiting at baseline (i.e. before the study) |
| Hou HK 2011 | Not eligible comparator: acupuncture vs. active control (metoclopramide) |
| Hou JY 2015 | Not eligible study aim: irrelevant study aim, and no nausea/vomiting outcome reported |
| Hu GW 2016 | Not eligible study aim: irrelevant study aim, and no nausea/vomiting outcome reported |
| Huang HX 2013 | Not eligible study aim: irrelevant study aim, and no nausea/vomiting outcome reported |
| Huang XM 1994 | Not eligible intervention: traditional Chinese medicine (patent medicine) |
| Jia YJ 2008 | Not eligible intervention: herbal medicine |
| Jiang S 2020 | Not eligible study aim: irrelevant study aim, and no nausea/vomiting outcome reported |
| Jiang SJ 2011 | Not eligible intervention: acupuncture, psychological care |
| Kang 2019 | Not eligible study design: animals experiment |
| Kim 2013 | Not eligible study aim: irrelevant study aim, and no nausea/vomiting outcome reported (Protocol of an excluded study - Kim 2016) |
| Kim 2016 | Not eligible study aim: irrelevant study aim, and no nausea/vomiting outcome reported |
| Lai ML 2015 | Not eligible population: all patients were experiencing chemotherapy induced nausea and/or vomiting at baseline (i.e. before the study) |
| Li 1998 | Not eligible population: radiochemotherapy |
| Li 2012 | Not eligible intervention: Chinese medicine |
| LI D 2002 | Not eligible study design: not randomized controlled trial |
| Li DQ 2012 | Not eligible study aim: irrelevant study aim, and no nausea/vomiting outcome reported |
| Li F 2018 | Not eligible study aim: irrelevant study aim, and no nausea/vomiting outcome reported |
| Li H 1997 | Not eligible population: radiochemotherapy |
| Li JG 2016 | Not eligible study aim: irrelevant study aim, and no nausea/vomiting outcome reported |
| Li QW 2016 | Not eligible population: patients were experiencing nausea and/or vomiting at baseline (i.e. before the study) (Multi report of an excluded study - LI QW 2020) |
| Li QW 2017 | Not eligible population: patients were experiencing nausea and/or vomiting at baseline (i.e. before the study) (Conference abstract of an excluded study - LI QW 2020) |
| Li QW 2017 | Not eligible population: patients were experiencing nausea and/or vomiting at baseline (i.e. before the study) (Protocol of an excluded study - LI QW 2020) |
| Li QW 2020 | Not eligible population: patients were experiencing nausea and/or vomiting at baseline (i.e. before the study) |
| Li SY 2018 | Not eligible study aim: irrelevant study aim, and no nausea/vomiting outcome reported |
| Li YK 2012 | Not eligible intervention: herbal medicine |
| Lian WL 2015 | Not eligible intervention: herbal medicine |
| Liao LL 2019 | Not eligible study aim: irrelevant study aim, and no nausea/vomiting outcome reported |
| Lin GH 2010 | Not eligible study aim: irrelevant study aim, and no nausea/vomiting outcome reported |
| Lin JS 2018 | Not eligible intervention: acupuncture was administered after the chemotherapy, thus it's not likely for the prevention of CINV |
| Liu D 2014 | Not eligible population: all patients were experiencing chemotherapy induced nausea and/or vomiting at baseline (i.e. before the study) |
| Liu SD 2017 | Not eligible study aim: irrelevant study aim, and no nausea/vomiting outcome reported |
| Liu Y 2006 | Not eligible population: no chemotherapy scheduled. Undergoing surgery |
| Liu Y 2015 | Not eligible intervention: ginger moxibustion |
| Liu Z 2018 | Not eligible study aim: irrelevant study aim, and no nausea/vomiting outcome reported |
| LU 2016 | Not eligible population: radiochemotherapy |
| Lu D 2017 | Not eligible intervention: electrothermal acupuncture |
| Lu DR 2015 | Not eligible study design: not randomized controlled trial |
| Lu W 2009 | Not eligible study aim: irrelevant study aim, and no nausea/vomiting outcome reported |
| Lu W 2010 | Not eligible population: radiochemotherapy |
| LU W 2012 | Not eligible intervention: acupuncture was administered after the chemotherapy, thus it's not likely for the prevention of CINV |
| Lu W 2013 | Not eligible study aim: irrelevant study aim, and no nausea/vomiting outcome reported |
| Lu W 2016 | Not eligible study aim: irrelevant study aim, and no nausea/vomiting outcome reported |
| Lu W 2017 | Not eligible study aim: irrelevant study aim, and no nausea/vomiting outcome reported |
| Lu W 2020 | Not eligible study aim: irrelevant study aim, and no nausea/vomiting outcome reported |
| Lu Y 2005 | Not eligible study aim: irrelevant study aim, and no nausea/vomiting outcome reported |
| Luo JQ 2009 | Not eligible intervention: herbal medicine |
| Ma TT 2009 | Not eligible study design: not randomized controlled trial |
| Ma Y 2016 | Not eligible study design: not randomized controlled trial |
| Mehling 20107 | Not eligible intervention: massage |
| Molasiotis 2019 | Not eligible study aim: irrelevant study aim, and no nausea/vomiting outcome reported |
| Molassiotis 2020 | Not eligible study aim: irrelevant study aim, and no nausea/vomiting outcome reported |
| Ni WM 2018 | Not eligible intervention: cupping |
| P.C.Leung 2004 | Not eligible study design: not randomized controlled trial |
| Park 2016 | Not eligible study aim: irrelevant study aim, and no nausea/vomiting outcome reported |
| Peng GY 2016 (1) | Not eligible population: radiochemotherapy |
| Peng GY 2016 (2) | Not eligible study aim: irrelevant study aim, and no nausea/vomiting outcome reported |
| Piedalue 2019 | Not eligible population: no chemotherapy scheduled |
| Price 2006 | Not eligible study design: not randomized controlled trial |
| Qing P 2020 | Not eligible study aim: irrelevant study aim, and no nausea/vomiting outcome reported |
| Quinlan 2016 | Not eligible population: no chemotherapy scheduled. Undergoing surgery |
| Reindl 2006 | Not eligible population: children |
| Ren JJ 2013 | Not eligible study aim: irrelevant study aim, and no nausea/vomiting outcome reported |
| Rithirangsriroj 2015 | Not eligible comparator: acupuncture vs. active control (ondansetron) |
| Roscoe 2002 | Not eligible intervention: acustimulation wristbands |
| Rostock 2013 | Not eligible study aim: irrelevant study aim, and no nausea/vomiting outcome reported |
| Salmon 2013 | Not eligible study design: not randomized controlled trial |
| Saraswati 2019 | Not eligible intervention: acupuncture was administered after the chemotherapy, thus it's not likely for the prevention of CINV |
| Sawada 2010 | Not eligible study design: not randomized controlled trial |
| Schroeder 2012 | Not eligible study aim: irrelevant study aim, and no nausea/vomiting outcome reported |
| Sevil 2016 | Not eligible intervention: acupressure, acupuncturepoint injection |
| Shen GW 2001 | Not eligible study aim: irrelevant study aim, and no nausea/vomiting outcome reported |
| Shen Y 2015 | Not eligible intervention: non-invasive electrostimulation |
| Shi Y 2020 | Not eligible study aim: irrelevant study aim, and no nausea/vomiting outcome reported |
| Sima L 2009 | Not eligible study design: paired, cross-controlled study design. Contacted the author, no response received |
| Sun L 2020 | Not eligible study aim: irrelevant study aim, and no nausea/vomiting outcome reported |
| Tan C 2015 | Not eligible population: radiotherapy |
| Tan XY 2019 | Not eligible study aim: irrelevant study aim, and no nausea/vomiting outcome reported |
| Tas 2014 | Not eligible study design: not randomized controlled trial |
| Tian X 2017 | Not eligible comparator: comparison between different expertise of acupuncturists |
| Tong 2018 | Not eligible study aim: irrelevant study aim, and no nausea/vomiting outcome reported |
| Varejão 2019 | Not eligible population: children |
| Vickers 2001 | Not eligible study design: not randomized controlled trial |
| Vickers 2004 | Not eligible study design: not randomized controlled trial |
| Walker 2010 | Not eligible population: no chemotherapy scheduled |
| Wang 2015 | Not eligible comparator: comparison between different manipulation techniques |
| Wang B 2011 | Not eligible study aim: irrelevant study aim, and no nausea/vomiting outcome reported |
| Wang C 2016 | Not eligible comparator: comparison between different expertise of acupuncturepuncturists |
| Wang F 2017 | Not eligible comparator: acupuncture vs. active control (Ricotine and Squalene Tablets) |
| Wang N 2018 | Not eligible population: no chemotherapy scheduled |
| Wang T 2018 | Not eligible population: no chemotherapy scheduled |
| Wang XP 2006 | Not eligible study aim: irrelevant study aim, and no nausea/vomiting outcome reported |
| Wang Y 2016 | Not eligible intervention: nursing care |
| Wei SW 2016 | Not eligible study aim: irrelevant study aim, and no nausea/vomiting outcome reported |
| Widgren Y 2017 | Not eligible population: radiochemotherapy |
| Wong 2015 | Not eligible intervention: transcutaneous electrical nerve stimulation |
| Wu GL 2018 | Not eligible study aim: irrelevant study aim, and no nausea/vomiting outcome reported |
| Wu Q 2019 | Not eligible population: no chemotherapy scheduled |
| Wu Y 2018 | Not eligible study aim: irrelevant study aim, and no nausea/vomiting outcome reported |
| Xia 2017 | Not eligible comparator: comparison between different expertise of acupuncturists |
| Xie 2017 | Not eligible intervention: transcutaneous electrical nerve stimulation |
| Xie D 2007 | Not eligible intervention: acupuncture was administered after the chemotherapy, thus it's not likely for the prevention of CINV |
| Xie Q 2010 | Not eligible population: radiotherapy |
| Xie T 2018 | Not eligible intervention: traditional Chinese medicine (patch) |
| Xu WR 2008 | Not eligible study aim: irrelevant study aim, and no nausea/vomiting outcome reported |
| Xue WX 2011 | Not eligible study aim: irrelevant study aim, and no nausea/vomiting outcome reported |
| XuY 2016 | Not eligible comparator: acupuncture vs. active control (Domperidone) |
| Yan JH 2018 | Not eligible intervention: herbal medicine |
| Yang HL 2005 | Not eligible intervention: traditional Chinese medicine (injection) |
| Yang HP2020 | Not eligible study aim: irrelevant study aim, and no nausea/vomiting outcome reported |
| Yang Y 2009 | Not eligible population: patients were experiencing nausea and/or vomiting at baseline (i.e. before the study) |
| Yared 2013 | Not eligible intervention: transcutaneous electrical nerve stimulation |
| Yeh 2012 | Not eligible population: children |
| Yin JJ 2015 | Not eligible study aim: irrelevant study aim, and no nausea/vomiting outcome reported |
| Yu C 2015 | Not eligible population: no chemotherapy scheduled |
| Yu MW 2017 | Not eligible population: no chemotherapy scheduled |
| Zeng 2014 | Not eligible study aim: irrelevant study aim, and no nausea/vomiting outcome reported |
| Zhai YQ 2018 | Not eligible study aim: irrelevant study aim, and no nausea/vomiting outcome reported |
| Zhang C 2018 | Not eligible study aim: irrelevant study aim, and no nausea/vomiting outcome reported |
| Zhang S 2017 | Not eligible study aim: irrelevant study aim, and no nausea/vomiting outcome reported |
| Zhang SQ 2017 | Not eligible study aim: irrelevant study aim, and no nausea/vomiting outcome reported |
| Zhang WW 2018 | Not eligible intervention: acupuncture was administered after the chemotherapy, thus it's not likely for the prevention of CINV |
| Zhang Z 2020 | Not eligible study aim: irrelevant study aim, and no nausea/vomiting outcome reported |
| Zhang ZY 2018 | Not eligible intervention: umbilical application |
| Zhao 2001 | Not eligible study aim: irrelevant study aim, and no nausea/vomiting outcome reported |
| Zhao CT 2016 | Not eligible population: no chemotherapy scheduled |
| Zhao H 2017 | Not eligible study aim: irrelevant study aim, and no nausea/vomiting outcome reported |
| Zhao WP 2015 | Not eligible study aim: irrelevant study aim, and no nausea/vomiting outcome reported |
| Zheng WL 2019 | Not eligible intervention: traditional Chinese medicine (patent medicine) |
| Zhong H 2016 | Not eligible study aim: irrelevant study aim, and no nausea/vomiting outcome reported |
| Zhong Y 2015 | Not eligible population: radiotherapy |
| Zhou 1999 | Not eligible population: radiochemotherapy |
| Zhou H 2003 | Not eligible study aim: irrelevant study aim, and no nausea/vomiting outcome reported |
| Zhou HZ 1996 | Not eligible study aim: irrelevant study aim, and no nausea/vomiting outcome reported |
| Zhou J 2017 | Not eligible population: patients were experiencing nausea and/or vomiting at baseline (i.e. before the study) |
| Zhou JQ 2004 | Not eligible intervention: acupressure, acupuncturepoint injection |
| Zhou Q 2017 | Not eligible intervention: Weitan Waifu patch |
| Zhu DL 2016 | Not eligible study aim: irrelevant study aim, and no nausea/vomiting outcome reported |
| Zhu SH 2010 | Not eligible study aim: irrelevant study aim, and no nausea/vomiting outcome reported |
| Zhu Z 2017 | Not eligible study aim: irrelevant study aim, and composite outcome. Contacted the author, no response received |
