# Supplementary material for: Acupuncture for the prevention of chemotherapy‐induced nausea and vomiting in cancer patients: A systematic review and meta‐analysis
Source: Cancer Med. 2023 May 24;12(11):12504–17. doi: 10.1002/cam4.5962 (PMC10278514; doi:10.1002/cam4.5962)
Supplement: Supplementary file 6 — Appendix S6 [file CAM4-12-12504-s002.docx]

Appendix 6. Descriptive Summary of Included Studies

| **Author and year (included in MA)** | **Study design:**  a. Country  b. Type of RCT, no. of arms, no. centers  c. Clinical setting  d. Sample size (I/C1/C2) | **Patients characteristics:**  a. Male (%)  b. Age (Years)  c. Cancer diagnose and stage  d. Previous chemotherapy experience (%) | **Chemotherapy treatment:**  a. Chemotherapy regimen  b. Emetic risk level of chemotherapy | **Interventions:**  a. Comparison type  b. UC medication  c. ACU rational  d. No. of needles and acupoint names  e. Needle stimulation, depth of insertion, response sought, and needle retention time (minutes)  f. ACU treatment regimen  g. ACU practitioner background  h. Initial ACU timepoint as compared with the start of chemotherapy |
| --- | --- | --- | --- | --- |
| Beith JM et al 2012 (Yes) | a. Australia  b. Parallel, two-armed, single center  c. NR  d. 32 (15/17) | a. 0  b. Mean (SD): 52 (9)  c. Breast cancer, early stage  d. 0 | a. Patients receive one of the following regimen:  1. 40% of patients - combination of Adriamycin (60mg/m2) and Cyclophosphamide (600 mg/m^2^);  2. 60% of patients - combination of Epirubicin (100 mg/m2), Cyclophosphamide (500 mg/m2), and 5-Fluorouracil (500 mg/m2)  b. Moderate | a. ACU and UC vs. Sham ACU and UC  b. Tropisetron, Aprepitant, Dexamethasone  c. TCM acupuncture; Fixed prescription  d. 4 needles: PC6 (uni - left on day 1, right on day 2); LI4 (uni - right on day 1, left on day 2); ST36 (bilateral)  e. EA; NR; De Qi response sought; 20  f. Once per day, two treatment sessions  g. Acupuncturists with more than 6 years of training and 10 years of experience  h. Prior to the start of chemotherapy |
| Brinkhaus B et al 2019 (No) | a. Germany  b. Parallel, two-armed, single center  c. NR  d. 150 (75/75) | a. 0  b. Mean (SD): 51 (10)  c. Breast cancer, T2 stage  d. 0 | a. Patients receive one of the following regimen:  1. 35.33% of patients - Epirubicin/Cyclophosphamid/Paclitaxel  2. 21.33% of patients - Epirubicin/Cyclophosphamid/Docetaxel  3. 20.00% of patients - 5-Fluorourazyll/Epirubicin/Cyclophosphamid  4. 17.33% of patients - 5-Fluorourazyll/Epirubicin/Cyclophosphamid/Docetaxel  5. 6.00% of patients - Epirubicin/Cyclophosphamid/Trastzumab  b. Moderate or High | a. ACU and UC vs. UC  b. NR  c. TCM acupuncture; Personalized prescription  d. NR needles: PC6; ST36; ST44; CV12; CV10  e. MA; NR; NR; Not predefined  f. NR  g. TCM practitioner with more than 30 years experience in acupuncture  h. Same time with chemotherapy |
| Cai LH et al 2011 (Yes) | a. China  b. Parallel, two-armed, single center  c. Inpatient  d. 80 (38/42) | a. 71  b. Mean (SD): 63.35 (15.18), Range: 48 - 78  c. Lung cancer, NR  d. NR | a. All patients receive: combination of Gemcitabine and Cisplatin  b. High | a. ACU and UC vs. UC  b. Ondansetron, Metoclopramide, Dexamethasone  c. TCM acupuncture; Fixed prescription  d. 2 needles: ST36 (bilateral)  e. MA; NR; De Qi response sought; 30  f. Once per day, five treatment sessions  g. NR  h. NR |
| Cao HQ et al 2006 (No) | a. China  b. Cross-over, two-armed, single center  c. NR  d. 26 (NR/NR) | a. 58  b. Mean (SD): 51.5 (4.2), Range: 38 - 72  c. Lung cancer, NR  d. 92 | a. All patients receive: Vinorelbine and Cisplatin  b. High | a. ACU and UC vs. UC  b. Ondansetron Hydrochloride, Dexamethasone  c. TCM acupuncture; Fixed prescription  d. 2 needles: ST36 (uni); CV12  e. MA; NR; De Qi response sought; 30  f. Twice per day, six treatment sessions  g. NR  h. NR |
| Chen C et al 2004 (No) | a. China  b. Parallel, two-armed, single center  c. NR  d. 56 (28/28) | a. 68  b. Mean (SD): NR  c. Non-small cell lung cancer, NR  d. NR | a. Patients receive the following regimen based on the cancer type:  1. Nasopharyngeal cancer uses: Cisplatin 60mg/m2, day1, Fluorouracil 500mg/2, day1-day5, Cyclophosphamide 600mg/m2, day1-day2, every 21 Day is 1 cycle;  2. Non-small cell carcinoma: Cisplatin 60mg/m2, day1, Etoposide 90-100mg/m2, day1-day3, every 21 days is a cycle.  b. High | a. ACU and UC vs. UC  b. NR  c. TCM acupuncture; Fixed prescription  d. 2 needles: ST36 (bilateral)  e. EA; NR; De Qi response sought; 20  f. Once per day, unclear treatment session  g. NR  h. Prior to the start of chemotherapy |
| Chen M et al 2016 (No) | a. China  b. Parallel, two-armed, single center  c. Inpatient  d. 132 (66/66) | a. 68  b. Mean (SD): NR, Range: 38 - 82  c. Mixed cancer, NR  d. NR | a. All patients receive the following regimen: Oxaliplatin 90 mg/m2 added 5% Glucose 150mL intravenously for 2h, Leucovorin 200mg/m2 added 5% Glucose 250mL intravenously 2 hours, after Cisplatin and Fluorouracil instillation, 600 mg/m2 of 5-Fluorouracil will be administered intravenously with 800 ml of 5% Glucose for 8-10 hours, 6 days is a course of treatment.  b. Moderate | a. ACU and UC vs. UC  b. Granisetron hydrochloride  c. TCM acupuncture; Fixed prescription  d. 6 needles: PC6 (bilateral); ST36 (bilateral); ST25 (bilateral)  e. Both MA and EA; NR; De Qi response sought; NR  f. Twice per day, unclear treatment session  g. Acupuncturists  h. NR |
| Chen RB et al 2011 (Yes) | a. China  b. Parallel, two-armed, single center  c. Inpatient  d. 51 (25/26) | a. 47  b. Mean (SD): NR  c. Lung cancer, NR  d. 63 | a. Patients receive different chemotherapy regimens according to different types of tumors and clinical conditions: Combination chemotherapy based on Cisplatin, Acetaminophen, Oxaliplatin, Gemcitabine, Cyclophosphamide, Vincristine, 5-Fluorouracil, etc. were selected respectively.  b. Moderate or High | a. ACU and UC vs. UC  b. Patients receive one of the following medication: 1. Ondansetron; or 2. Granisetron Hydrochloride.  c. TCM acupuncture; Fixed prescription  d. 7 needles: CV12 (bilateral); PC6 (bilateral); CV12; SP4 (bilateral)  e. Both MA and EA; Deep needling (>=10 mm); De Qi response sought; 30  f. Once per day, unclear treatment session  g. NR  h. Prior to the start of chemotherapy |
| Dundee JW et al 1987 (No) | a. United Kingdom  b. NR, two-armed, NR  c. Inpatient  d. 10 (NR/NR) | a. NR  b. Mean (SD): NR  c. Testicular cancer, NR  d. 100 | a. All patients receive infusion containing Cisplatin 30mg as part of a regimen.  b. High | a. ACU and UC vs. Sham ACU and UC  b. NR  c. TCM acupuncture; Fixed prescription  d. NR needles: PC6  e. EA; NR; NR; NR  f. NR  g. NR  h. NR |
| Guo JY et al 2016 (Yes) | a. China  b. Parallel, two-armed, single center  c. NR  d. 60 (NR/NR) | a. NR  b. Mean (SD): NR  c. NR, NR  d. NR | a. All patients receive: Cisplatin (75mg/m2, the first and second days)  b. High | a. ACU and UC vs. UC  b. Tropisetron, Dexamethasone  c. Wrist ankle acupuncture; Fixed prescription  d. NR needles: Needle at the wrist about 2 fingers upon the transverse crease of the wrist, and the ankle about 3 fingers upon the ankle joint  e. MA; NR; No response sought; 30  f. Once per day, five treatment sessions  g. NR  h. Prior to the start of chemotherapy |
| Han Y et al 2018 (No) | a. China  b. Parallel, two-armed, single center  c. NR  d. 112 (56/56) | a. 54  b. Mean (SD): NR  c. Mixed cancer, NR  d. NR | a. NR  b. Unclear | a. ACU and UC vs. UC  b. Granisetron hydrochloride  c. TCM acupuncture; Fixed prescription  d. 6 needles: PC6 (bilateral); ST36 (bilateral); ST25 (bilateral)  e. Both MA and EA; NR; De Qi response sought; NR  f. Twice per day, six treatment sessions  g. Acupuncturists with more than 10 years of experience  h. NR |
| Huang KJ et al 2016 (Yes) | a. China  b. Parallel, two-armed, two centers  c. Outpatient  d. 120 (60/60) | a. 0  b. Range: 35 - 65  c. Breast cancer, III-IV stage  d. 0 | a. All patients receive: 5-Fluorouracil, Doxorubicin and Cyclophosphamide.  b. Moderate | a. ACU and UC vs. UC  b. Granisetron hydrochloride  c. TCM acupuncture; Personalized prescription  d. NR needles: (as main acupoints) CV12; CV11; ST36, ST40; PC6  e. Both MA and EA; Varied; NR; 30  f. Twice per day, eight treatment sessions  g. NR  h. Prior to the start of chemotherapy |
| Huang ZF et al 2008 (No) | a. China  b. Parallel, two-armed, NR  c. Inpatient  d. 80 (40/40) | a. 75  b. Mean (SD): NR  c. Lung cancer, NR  d. 41 | a. Patients receive cisplatin-based regimen, according to cancer type:  1. Non-small cell lung cancer, uses Cisplatin and Fluorouracil, or Cisplatin and Gaynor regimen;  2. Nasopharyngeal carcinoma, uses Fludarabine and Cyclophosphamide regimen;  3. Esophageal cancer, uses Cisplatin and Fluorouracil regimen.  b. High | a. ACU and UC vs. UC  b. Granisetron hydrochloride, Dexamethasone  c. TCM acupuncture; Fixed prescription  d. 4 needles: PC6 (bilateral); ST36 (bilateral)  e. MA; NR; De Qi response sought; 30  f. Once per day, five treatment sessions  g. NR  h. NR |
| Jiao DP et al 2008 (No) | a. China  b. Parallel, two-armed, single center  c. Inpatient  d. 66 (34/32) | a. 55  b. Mean: 57, Range: 42 - 72  c. Mixed cancer, III stage  d. NR | a. Patients receive different chemotherapy regimens according to different types of tumors:  1. Lung cancer, uses Docetaxel and Cisplatin regimen;  2. Liver cancer, uses trans-arterial chemoembolization;  3. Gastric cancer, uses Oxaliplatin plus Leucovorin and 5-Fluorouracil regimen;  4. Gallbladder cancer and pancreatic cancer, uses Aidi injection (Guizhou Yibai) biological chemotherapy and thymosin immunotherapy.  b. Moderate | a. ACU and UC vs. UC  b. Pain relief medication  c. TCM acupuncture; Personalized prescription  d. NR needles: Bei Shu points according to different organs/cancer types and/or the location of pain; Xi-Cleft points according to the location of pain; ST36  e. MA; NR; De Qi response sought; 30  f. Once per day, seven treatment sessions  g. NR  h. NR |
| Lai HK et al 2011 (No) | a. China  b. Parallel, two-armed, single center  c. Inpatient  d. 60 (30/30) | a. 45  b. Range: 32 - 70  c. Mixed cancer, NR  d. NR | a. All patients receive: chemotherapy regimen containing 40mg/m 2 of cisplatin, intravenous drip on day 1~3.  b. High | a. ACU and UC vs. UC  b. Tropisetron  c. TCM acupuncture; Fixed prescription  d. 6 needles: PC6 (bilateral); ST36 (bilateral); SP4 (bilateral)  e. MA; NR; De Qi response sought; 30  f. Once per day, six treatment sessions  g. NR  h. NR |
| Li D et al 2017 (Yes) | a. China  b. Parallel, two-armed, single center  c. Inpatient  d. 40 (20/20) | a. 0  b. Mean (SD): NR  c. Breast cancer, NR  d. 100 | a. Patients receive Anthracycline-containing chemotherapy regimens:  1. 50% patients: Epirubicin, Cyclophosphamide;  2. 50% patients: Fluorouracil, Epirubicin, Cyclophosphamide.  b. Moderate or High | a. ACU and UC vs. UC  b. Tropisetron hydrochloride  c. TCM acupuncture; Fixed prescription  d. 12 needles: ST36 (bilateral); SP6 (bilateral); ST25 (bilateral); CV6; CV4; LV3 (bilateral); PC6 (bilateral)  e. MA; Deep needling (>=10 mm); De Qi response sought; 30  f. NR  g. NR  h. Same time with chemotherapy |
| Li ML et al 2016 (No) | a. China  b. Cross-over, two-armed, single center  c. Inpatient  d. 40 (20/20) | a. 48  b. Range: 20 - 76  c. Lung cancer, NR  d. 100 | a. Patients receive one of the following:  1. Adriamycin and Cyclophosphamide;  2. Etoposide and Cisplatin;  3. Taxanes and Cisplatin;  4. Docetaxel and Cisplatin;  5. Oxaliplatin plus Leucovorin and 5-Fluorouracil.  In addition, this study clarified "50% of patients receive High Emetic Risk chemotherapy"  b. Moderate or High | a. ACU and UC vs. UC  b. Tropisetron hydrochloride, metoclopramide  c. TCM acupuncture; Fixed prescription  d. 4 needles: CV12; CV10; CV6; CV4  e. MA; Deep needling (>=10 mm); No response sought; 30  f. NR  g. Acupuncturist with 5 years experience  h. Prior to the start of chemotherapy |
| Li QL et al 2007 (No) | a. China  b. Parallel, two-armed, single center  c. Inpatient  d. 63 (33/30) | a. 48  b. Range: 23 - 73  c. Mixed cancer, NR  d. 100 | a. Patients receive chemotherapy according to the cancer types and pathological typings:  Cisplatin, Taxotere, Gemcitabine, Oxaliplatin, Cyclophosphamide, Vincristine, 5-Fluorouracil.  b. Moderate or High | a. ACU and UC vs. UC  b. Ondansetron, Dexamethasone  c. TCM acupuncture; Fixed prescription  d. 2 needles: BL17 (bilateral)  e. EA; Deep needling (>=10 mm); De Qi response sought; 20  f. Once per day, seven treatment sessions  g. NR  h. NR |
| Li YH et al 2014 (No) | a. China  b. Parallel, two-armed, single center  c. Inpatient  d. 72 (36/36) | a. 69  b. Range: 45 - 71  c. Lung cancer, III-IV stage  d. NR | a. All patients receive Gemcitabine plus Cisplatin regimen: Gemcitabine will be administered 1000 mg/m2 intravenously (30-60 min, inf) on day 1 and 8; Cisplatin day 1~3 30 mg/m2 intravenous injection (30~60 min, inf), proper hydration before treatment; repeat 1 cycle every 21 days for a total of 2 cycles.  b. Moderate or High | a. ACU and UC vs. UC  b. Recombinant human granulocyte colony-stimulating factor (rhG-CSF)  c. TCM acupuncture; Fixed prescription  d. NR needles: GV14; BL17; ST36  e. MA; NR; De Qi response sought; 30  f. Twice per day, ten treatment sessions  g. NR  h. Prior to the start of chemotherapy |
| Liao GY et al 2018 (No) | a. China  b. Parallel, two-armed, single center  c. NR  d. 59 (29/30) | a. 80  b. Range: 21 - 75  c. Lung cancer, NR  d. NR | a. All patients receive Platinum-based chemotherapy.  b. Moderate or High | a. ACU and UC vs. UC  b. NR  c. Xuan Ji acupuncture; Fixed prescription  d. 6 needles: Small Heavenly Heart (left); ST36 (right); T33.12 (right); SJ6 (right); LV3 (left); SP3 (left)  e. MA; NR; De Qi response sought; 30  f. Once per day, five treatment sessions  g. NR  h. NR |
| Liu M et al 2017 (Yes) | a. China  b. Parallel, two-armed, single center  c. NR  d. 63 (32/31) | a. 55  b. Mean (SD): NR  c. Colon cancer, NR  d. NR | a. All patients receive: Oxaliplatin 85 mg/m2 + Leucovorin 200 mg/m2 + 5-Fluorouracil 400 mg/m2, the medicinal route is intravenous infusion for 2 hours, followed by 5-Fluorouracil 600mg/m2 intravenous bolus, and continuous chemotherapy for 2 days.  b. Moderate | a. ACU and UC vs. UC  b. Tropisetron hydrochloride  c. TCM acupuncture; Fixed prescription  d. 6 needles: CV6; CV4; PC6 (bilateral); ST36 (bilateral)  e. MA; Deep needling (>=10 mm); De Qi response sought; 30  f. Once per day, five treatment sessions  g. NR  h. Prior to the start of chemotherapy |
| Lv JS et al 2012 (No) | a. China  b. Parallel, two-armed, single center  c. Inpatient  d. 60 (30/30) | a. 52  b. Range: 23 - 70  c. Mixed cancer, NR  d. NR | a. NR  b. Unclear | a. ACU and UC vs. UC  b. Granisetron  c. TCM acupuncture; Fixed prescription  d. 5 needles: CV12; PC6 (bilateral); ST36 (bilateral)  e. MA; Deep needling (>=10 mm); De Qi response sought; 30  f. Once per day, seven treatment sessions  g. NR  h. Prior to the start of chemotherapy |
| McKeon C et al 2015 (No) | a. Australia  b. Parallel, three-armed, single center  c. NR  d. 40 (21/19/20) | a. 12  b. Range: 27 - 77, Median (IQR): 57.5 (15)  c. Breast cancer, NR  d. 0 | a. NR  b. High | a. ACU and UC vs. Sham ACU and UC vs. UC  b. Regimen1. Ondansetron, Dexamethasone  Regimen2. Ondansetron, Dexamethasone, aprepitant  c. TCM acupuncture; Fixed prescription  d. 8 needles: ST36 (bilateral); PC6 (bilateral); LV3 (bilateral); LI4 (bilateral);  e. EA; Deep needling (>=10 mm); De Qi response sought; 30  f. Once per day, two treatment sessions  g. Acupuncturists with an advanced diploma of acupuncture and with 6 years of experience  h. Prior to the start of chemotherapy |
| Qi J et al 2018 (No) | a. China  b. Parallel, two-armed, single center  c. Inpatient  d. 60 (30/30) | a. 52  b. Range: 40 - 75  c. NR, NR  d. NR | a. NR  b. Unclear | a. ACU and UC vs. UC  b. Granisetron  c. TCM acupuncture; Fixed prescription  d. 7 needles: PC6 (bilateral); ST36 (bilateral); SP6 (bilateral); CV12  e. MA; NR; De Qi response sought; 30  f. Twice per day, unclear treatment session  g. NR  h. Prior to the start of chemotherapy |
| Shen BY et al 2013 (No) | a. China  b. Parallel, two-armed, two centers  c. Inpatient  d. 60 (30/30) | a. 55  b. Range: 35 - 71  c. Mixed cancer, NR  d. NR | a. All patients receive FOLFOX4 regimen, no detail reported.  b. Moderate | a. ACU and UC vs. UC  b. Granisetron hydrochloride  c. TCM acupuncture; Fixed prescription  d. NR needles: PC6; ST36  e. MA; NR; De Qi response sought; NR  f. Twice per day, unclear treatment session  g. Acupuncturists  h. NR |
| Shen J et al 2000 (No) | a. United States  b. Parallel, three-armed, single center  c. Inpatient  d. 70 (37/33/34) | a. 0  b. Mean: 46  c. Breast cancer, NR  d. 100 | a. On hospital days 1, 2, and 3, all patients received high doses of Cyclophosphamide and Cisplatin, and on day 4, Carmustine.  Administration of the chemotherapy: Cyclophosphamide, 1875 mg/m2 body-surface area, per day over 60 minutes, starting at 9 AM for 3 days; Cisplatin, 55 mg/m2 body-surface area, per day with continuous infusion, starting at 9 AM for 3 days; Carmustine, 600 mg/m2 body-surface area, over 2 hours, starting at 9 am, immediately after the cisplatin dose was completed.  b. High | a. ACU and UC vs. UC  b. Prochlorperazine, Lorazepam, Diphenhydramine hydrochloride  c. TCM acupuncture; Fixed prescription  d. 4 needles:  e. EA ; Deep needling (>=10 mm); De Qi response sought; 30  f. Once per day, five treatment sessions  g. A clinical instructor at the medical school and had 3 years of acupuncture training, and an acupuncture clinician with 20 years of practicing experience.  h. Prior to the start of chemotherapy |
| Streitberger K et al 2003 (Yes) | a. Germany  b. Parallel, two-armed, single center  c. NR  d. 80 (41/39) | a. 51  b. Mean (SD): NR  c. NR, NR  d. 100 | a. Patients receive one of the following high-dose chemotherapy regimen:  1. mainly Melphalan (200 mg/m2, day 1), but also BEAM [BCNO(300 mg/m ), day 1; Cytosine Arabinoside (200 mg/m ) and Etoposide (100 mg/m ), day 2–5; Melphalan (140 mg/m ), day6]; Or  2. VIC [Ifosphamide (4000 mg/m2), Etoposide (500mg/m2), and Carboplatin (500 mg/m2), day 1–3], and interleukin converting enzyme [Ifosphamide (2400 mg/m2), Epirubicin (36 mg/m2), carboplatin (180 mg/m2), day 1–5].  None of the chemotherapy regimen included steroids.  b. High | a. ACU and UC vs. Sham ACU and UC  b. Ondansetron  c. TCM acupuncture; Fixed prescription  d. 2 needles: PC6 (bilateral)  e. MA; NR; De Qi response sought; 20  f. NR  g. Trained acupuncturists  h. Prior to the start of chemotherapy |
| Teng HQ et al 2007 (No) | a. China  b. Cross-over, two-armed, NR  c. NR  d. 40 (NR/NR) | a. 64  b. Range: 28 - 77  c. Lung cancer, NR  d. NR | a. Patients recieve Cisplatin, Doxorubicin, Cyclophosphatidylamine or other chemotherapy drugs.  b. Moderate or High | a. ACU and UC vs. UC  b. NR  c. TCM acupuncture; Fixed prescription  d. 6 needles: PC6 (bilateral); ST36(bilateral); LI4 (bilateral)  e. EA; NR; NR; 30  f. Unclear frequency per day, seven treatment sessions  g. NR  h. NR |
| Wang G et al 2016 (No) | a. China  b. Parallel, two-armed, single center  c. Inpatient  d. 60 (30/30) | a. 63  b. Mean (SD): NR  c. NR, NR  d. 0 | a. All patients receive Platinum-based chemotherapy.  b. Moderate or High | a. ACU and UC vs. UC  b. Tropisetron  c. Han Xuan acupuncture; Fixed prescription  d. 4 needles: T33 12 (right); ST36(right); LV3 (left); SP3 (left)  e. MA; NR; NR; 30  f. Once per day, seven treatment sessions  g. NR  h. NR |
| Wang JC et al 2019 (No) | a. China  b. Parallel, two-armed, single center  c. NR  d. 60 (30/30) | a. 77  b. Range: 40 - 76  c. Bowel cancer, NR  d. NR | a. NR  b. Unclear | a. ACU and UC vs. UC  b. Azasetron, Metoclopramide hydrochloride  c. Intradermal needle ; Fixed prescription  d. 2 needles: LI4 (left); PC6 (left)  e. MA; Superficial needling (<10 mm); De Qi response sought; NR  f. Not applicable frequency, unclear treatment session  g. Nurse  h. Prior to the start of chemotherapy |
| Wang YL et al 2019 (No) | a. China  b. Parallel, three-armed, single center  c. NR  d. 100 (50/50) in eligible comparator | a. 62  b. Mean (SD): NR  c. Lung cancer - Adenocarcinoma, NR  d. 100 | a. NR  b. Unclear | a. ACU and UC vs. UC  b. Tropisetron hydrochloride  c. TCM acupuncture; Fixed prescription  d. NR needles: PC6;ST36; CV12  e. MA; Deep needling (>=10 mm); De Qi response sought; 30  f. Once per day, three treatment sessions  g. NR  h. Prior to the start of chemotherapy |
| Wu BQ et al 2011 (Yes) | a. China  b. Parallel, two-armed, single center  c. Inpatient  d. 80 (38/42) | a. 71  b. Mean (SD): NR  c. Lung cancer, NR  d. NR | a. Patients receive one of the following regimen:  1. Gemcitabine-based regimens; Or  2. Vinorelbine-based regimens.  b. Low | a. ACU and UC vs. UC  b. Ondansetron hydrochloride, Metoclopramide, Dexamethasone  c. TCM acupuncture; Fixed prescription  d. 2 needles: ST36(bilateral)  e. MA; NR; De Qi response sought; 30  f. Once per day, five treatment sessions  g. NR  h. NR |
| Xu Y et al 2014 (Yes) | a. China  b. Parallel, two-armed, single center  c. Inpatient  d. 52 (27/25) | a. 58  b. Range: 20 - 80  c. Respiratory system tumors, NR  d. NR | a. Patients receive one of the following chemotherapy medicine:  1. Cisplatin; Or  2. Carboplatin; Or  3. Oxaliplatin; Or  4. Nidaplatin; Or  5. Non-platinum.  b. Moderate or High | a. ACU and UC vs. UC  b. Palonosetron Hydrochloride  c. Auricular acupuncture; Fixed prescription  d. 6 needles: Small intestine (bilateral); Stomach (bilateral); Esophagus (bilateral)  e. EA; NR; De Qi response sought; 30  f. Twice per day, three treatment sessions  g. NR  h. NR |
| Yan JH et al 2017 (No) | a. China  b. Parallel, two-armed, single center  c. Inpatient  d. 60 (30/30) | a. 58  b. Mean (SD): NR  c. NR, NR  d. NR | a. All patients receive Cisplatin.  b. High | a. ACU and UC vs. UC  b. Dexamethasone, Tropisetron  c. TCM acupuncture; Fixed prescription  d. 5needles: PC6 (bilateral);ST36 (bilateral); CV12  e. Both MA and EA; Deep needling (>=10 mm); No response sought; 30  f. Once per day, four treatment sessions  g. NR  h. Prior to the start of chemotherapy |
| Zhang J et al 2017 (Yes) | a. China  b. Cross-over, two-armed, single center  c. Inpatient  d. 40 (20/20) | a. 60  b. Mean (SD): 58 (9.4), Range: 34 - 73  c. Mixed cancer, IV stage  d. NR | a. Patients receive one of the following Platinum-based chemotherapy medicine:  1. Cisplatin; Or  2. Carboplatin; Or  3. Oxaliplatin; Or  4. Nidaplatin.  b. Moderate or High | a. ACU and UC vs. UC  b. Tropisetron mesylate, Metoclopramide hydrochloride  c. Xuan Ji acupuncture; Fixed prescription  d. 6needles: Small Heavenly Heart (left); ST36 (right); T33 12 (right); SJ6 (right); LV3 (left); SP3 (left)  e. MA; NR; NR; 30  f. Once per day, three treatment sessions  g. Acupuncturists  h. NR |
| Zhang LC et al 2014 (Yes) | a. China  b. Parallel, four-armed, single center  c. NR  d. 63 (33/30) | a. 48  b. Mean: 54, Range: 32 - 76  c. Mixed cancer, NR  d. 63 | a. All patients receive High-dose cisplatin (Cisplatinum diamine dichloride 80-100mg/m2) regimen chemotherapy.  b. High | a. ACU and UC vs. UC  b. Ondansetron  c. TCM acupuncture; Fixed prescription  d. NR needles: CV12; PC6; ST36  e. MA; NR; NR; 30  f. Twice per day, unclear treatment session  g. NR  h. Prior to the start of chemotherapy |
| Zhang X et al 2014 (Yes) | a. China  b. Parallel, two-armed, single center  c. Inpatient  d. 72 (38/34) | a. 60  b. Mean (SD): NR  c. Mixed cancer, NR  d. 100 | a. All patients receive One of the following: Cisplatin chemotherapy ≥ 60mg/m2, Carmustine >250mg/m2 or Cyclophosphamide or Dacarbazine >1 500 mg/m2, etc.  b. High | a. ACU and UC vs. Sham ACU and UC  b. Glatron hydrochloride  c. TCM acupuncture; Fixed prescription  d. 2 needles: PC6 (uni); PC5 (uni)  e. EA; Deep needling (>=10 mm); De Qi response sought; 60  f. Twice per day, three treatment sessions  g. NR  h. NR |
| Zhou L et al 2006 (No) | a. China  b. Cross-over, two-armed, NR  c. NR  d. 40 (NR/NR) | a. 68  b. Mean: 47.5, Range: 30 - 69  c. Mixed cancer, NR  d. 100 | a. Patients receive one of the following regimen:  1. small cell lung cancer: Etoposide and Cisplatin regimen, Etoposide, Ifosfamide, and Cisplatin regimen;  2. non-small cell lung cancer: Etoposide and Cisplatin regimen, Gemcitabine and Cisplatin regimen;  3. gastric cancer: Etoposide, Doxorubicin and Cisplatin regimen;  4. liver cancer: Cisplatin and 5-Fluorouracil regimen;  5. others are Cyclophosphamide, Adriamycin and Platinum regimens.  All regimens are cisplatin. The dose is 50~80mg/m2, the dose of Adriamycin, Tetrahydropyranyl Adriamycin is 40~50mg/m2, and the dose of Epirubicin is 80~100mg/m2.  b. Moderate | a. ACU and UC vs. UC  b. Granisetron  c. TCM acupuncture; Fixed prescription  d. NR needles: SP4; PC6; ST36  e. MA; NR; De Qi response sought; NR  f. Once or twice per day, unclear treatment session  g. NR  h. NR |
| Zhu WJ et al 2016 (Yes) | a. China  b. Parallel, two-armed, NR  c. Inpatient  d. 40 (21/19) | a. 65  b. Range: 27 - 80  c. Lung cancer, NR  d. NR | a. All patients receive Platinum-based chemotherapy.  b. Moderate or High | a. ACU and UC vs. UC  b. Palonosetron Hydrochloride  c. Auricular acupuncture; Fixed prescription  d. 4 needles: Small intestine (bilateral); Esophagus (bilateral)  e. EA; NR; NR; 30  f. Twice per day, three treatment sessions  g. NR  h. NR |

Note: Acupuncture points used: LI4 (Hegu), PC6 (Neiguan), PC5 (Jianshi), SP3 (Taibai), SP4 (Gongsun), SP6 (Sanyinjiao), SJ6 (Zhigou), LV3 (Taichong), ST36 (Zusanli), ST40 (Fenglong), ST44 (Neiting), ST25 (Tianshu), CV12 (Zhongwan), CV11 (Jianli), CV10 (Xiawan), CV6 (Qihai), CV4 (Guanyuan), GV14 (Dazhui), BL17 (Geshu), T33.12 (Xin Men), Xiao Tian Xin, Small intestine (auricular acupuncture - Xiaochang), Stomach (auricular acupuncture – Wei), Esophagus(auricular acupuncture - Shidao).

Abbreviations: MA, meta-analysis; RCT, randomized controlled trial; ACU, acupuncture; UC, usual care; MA, manual acupuncture; EA, electro-acupuncture; NR, not reported; SD, standard deviation; TCM, traditional Chinese medicine.
