# Supplementary material for: Acupuncture for the prevention of chemotherapy‐induced nausea and vomiting in cancer patients: A systematic review and meta‐analysis
Source: Cancer Med. 2023 May 24;12(11):12504–17. doi: 10.1002/cam4.5962 (PMC10278514; doi:10.1002/cam4.5962)
Supplement: Supplementary file 7 — Appendix S7 [file CAM4-12-12504-s011.docx]

| **Appendix 7. GRADE Summary of Findings Table** | | | | | |
| --- | --- | --- | --- | --- | --- |
| **Patient or population:** cancer patients schedule to receive chemotherapy  **Setting:** any  **Intervention:** acupuncture and usual care  **Comparison:** sham acupuncture and usual care | | | | | |
| **Outcomes** | **№ of participants (studies)** | **Certainty of the evidence (GRADE)** | **Relative effect (95% CI)** | **Anticipated absolute effects** | |
|  |  |  |  | **Risk with sham acupuncture and usual care** | **Risk difference with acupuncture and usual care** |
| Complete control of acute nausea and vomiting - not reported | - | **-** | - | - | - |
| Complete control of acute nausea | 110 (2 RCTs) | ⨁⨁◯◯  Low ^a, b^ | **RR 0.87** (0.26 to 2.90) | **Moderate risk** | |
|  |  |  |  | 50 per 100 ^c^ | **7 fewer per 100** (37 fewer to 95 more) |
| Complete control of acute vomiting | 182 (3 RCTs) | ⨁⨁◯◯ Low ^b, d, e^ | **RR 1.05** (0.72 to 1.53) | **Moderate risk** | |
|  |  |  |  | 50 per 100 ^c^ | **3 more per 100** (14 fewer to 27 more) |
| Complete control of delayed nausea and vomiting - not reported | - | **-** | - | - | - |
| Complete control of delayed nausea | 80 (1 RCT) | ⨁⨁◯◯ Moderate ^b, f^ | **RR 0.59** (0.27 to 1.26) | **Moderate risk** | |
|  |  |  |  | 50 per 100 ^c^ | **21 fewer per 100** (37 fewer to 13 more) |
| Complete control of delayed vomiting | 152 (2 RCTs) | ⨁◯◯◯ Very low ^b, d, g^ | **RR 1.10** (0.28 to 4.36) | **Moderate risk** | |
|  |  |  |  | 50 per 100 ^c^ | **5 more per 100** (36 fewer to 168 more) |
| Adverse events related to acupuncture | 112  (2 RCTs) | ⨁⨁◯◯ Moderate ^h^ | Two studies informed that there were no adverse events related to acupuncture. | | |
| ***The risk in the intervention group** (and its 95% confidence interval) is based on the assumed risk in the comparison group and the **relative effect** of the intervention (and its 95% CI).  **CI:** confidence interval; **RR:** risk ratio  **Thresholds for clinically important effects (benefit or worsening) based on the absolute risk difference:** Null effect: 0; Clinically irrelevant effect: lower than 10%; Small effect (clinically relevant): from 10% to less than 20%; Moderate effect: from 20% to less than 30%; Large effect: from 30%. | | | | | |
| **GRADE Working Group grades of evidence** **High certainty:** we are very confident that the true effect lies close to that of the estimate of the effect. **Moderate certainty:** we are moderately confident in the effect estimate: the true effect is likely to be close to the estimate of the effect, but there is a possibility that it is substantially different. **Low certainty:** our confidence in the effect estimate is limited: the true effect may be substantially different from the estimate of the effect. **Very low certainty:** we have very little confidence in the effect estimate: the true effect is likely to be substantially different from the estimate of effect. | | | | | |

**Explanations**

a. Downgraded due to imprecision by two levels. The 95% CI of the risk difference for the moderate risk scenario is compatible from a worsening of large magnitude to a benefit of large magnitude, including a null effect. In addition, the observed sample size is lower than the optimal information size (estimated at 816 patients, based on a basal risk of 50% and a relative effect of the intervention of 20%, RR=1.20).

b. As the meta-analysis included less than 10 studies, we were unable to detect publication bias.

c. Based on a network meta-analysis by Piechotta V et al. DOI: 10.1002/14651858.CD012775.pub2

d. Downgraded due to high risk of bias by one level because one of the study is at high risk of performance bias and detection bias, which might affect the interpretation of result.

e. Downgraded due to imprecision by one level. The 95% CI of the risk difference for the moderate risk scenario is compatible from a worsening of small magnitude to a benefit of moderate magnitude, including null effect. In addition, the observed sample size is lower than the optimal information size (estimated at 1,143 patients, based on a basal risk of 50% and a relative effect of the intervention of 20%, RR=1.20).

f. Downgraded due to imprecision by one level. The 95% CI of the risk difference for the moderate risk scenario is compatible from a worsening of large magnitude to a benefit of small magnitude, including null effect. In addition, the observed sample size is lower than the optimal information size (estimated at 1,275 patients, based on a basal risk of 50% and a relative effect of the intervention of 20%, RR=1.20).

g. Downgraded due to imprecision by two levels. The 95% CI of the risk difference for the moderate risk scenario is compatible from a worsening of large magnitude to a benefit of large magnitude, including null effect. In addition, the observed sample size is lower than the optimal information size (estimated at 854 patients, based on a basal risk of 50% and a relative effect of the intervention of 20%, RR=1.20).

h. Downgraded due to unclear risk of bias by one level because all studies were at unclear risk of performance bias, detection bias, and reporting bias.
