# Supplementary material for: Acupuncture for the prevention of chemotherapy‐induced nausea and vomiting in cancer patients: A systematic review and meta‐analysis
Source: Cancer Med. 2023 May 24;12(11):12504–17. doi: 10.1002/cam4.5962 (PMC10278514; doi:10.1002/cam4.5962)
Supplement: Supplementary file 8 — Appendix S8 [file CAM4-12-12504-s007.docx]

Appendix 8. Moderator Analysis for Complete Control of Acute Vomiting

| **Variables ^a^** | **No. of Studies** |  | **Effect Size and 95% Confidence Intervals** | | |  | **Test of Moderators (*p*)** |
| --- | --- | --- | --- | --- | --- | --- | --- |
|  |  |  | **RR ^b^** | **LL** | **UL** |  |  |
| **Sex** |  |  |  |  |  |  | .533 |
| Female | 1 |  | 1.14 | 0.80 | 1.64 |  | - |
| Mix | 8 |  | 1.25 | 1.13 | 1.38 |  | - |
| **Previous chemotherapy experience** |  |  |  |  |  |  | .488 |
| Mix | 2 |  | 1.10 | 0.85 | 1.42 |  | - |
| Yes | 1 |  | 1.14 | 0.80 | 1.64 |  | - |
| **Previous acupuncture experience** | 0 |  | - | - | - |  | - |
| **Emetic risk of chemotherapy** |  |  |  |  |  |  | .233 |
| High | 3 |  | 1.10 | 0.69 | 1.75 |  | - |
| Low | 1 |  | 1.47 | 0.96 | 2.25 |  | - |
| Moderate | 1 |  | 1.38 | 0.84 | 2.29 |  | - |
| Moderate or high | 5 |  | 1.19 | 1.06 | 1.34 |  | - |
| **Type of outcome measurement** | 0 |  | - | - | - |  | - |
| **Experience of intervention provider ≥ 5 years** | 0 |  | - | - | - |  | - |
| **Clinical setting** |  |  |  |  |  |  | - |
| Inpatient | 7 |  | 1.23 | 1.10 | 1.38 |  | - |
| **Allocation concealment** |  |  |  |  |  |  | .976 |
| Low risk of bias | 1 |  | 1.14 | 0.80 | 1.64 |  | - |
| Unclear risk of bias | 9 |  | 1.14 | 1.02 | 1.28 |  | - |
| **Attrition bias** |  |  |  |  |  |  | .357 |
| Low risk of bias | 3 |  | 1.21 | 0.89 | 1.66 |  | - |
| Unclear risk of bias | 7 |  | 1.10 | 0.96 | 1.27 |  | - |
| **Registration of study** | 0 |  | - | - | - |  | - |
| **Clinical design of RCT** |  |  |  |  |  |  | .218 |
| Cross-over | 1 |  | 1.32 | 1.03 | 1.69 |  | - |
| Parallel | 9 |  | 1.10 | 1.00 | 1.23 |  | - |
| **Study center** |  |  |  |  |  |  | - |
| Single center | 9 |  | 1.14 | 1.02 | 1.28 |  |  |
| **Reported as adequate training of intervention provider ^c^** |  |  |  |  |  |  | .218 |
| Unclear | 9 |  | 1.10 | 1.00 | 1.23 |  | - |
| Judged as adequately trained | 1 |  | 1.32 | 1.03 | 1.69 |  | - |
| **Rescue medication ^c^** |  |  |  |  |  |  | .035 |
| Unclear | 7 |  | 1.24 | 1.11 | 1.39 |  | - |
| Planned to administer additional antiemetics | 3 |  | 1.04 | 0.80 | 1.34 |  | - |

Notes: **a.** Variable categories are listed based on the availability of study-level information (i.e., if the numbers do not add up to the total number of studies, this is due to missing information); **b.** a RR > 1 indicates acupuncture increases the complete control rate, a RR < 1 indicates acupuncture decreases the complete control rate; **c.** Exploratory variable.

Abbreviations: RR, risk ratio; LL, lower limit; UL, upper limit.
