# Supplementary material for: Acupuncture for the prevention of chemotherapy‐induced nausea and vomiting in cancer patients: A systematic review and meta‐analysis
Source: Cancer Med. 2023 May 24;12(11):12504–17. doi: 10.1002/cam4.5962 (PMC10278514; doi:10.1002/cam4.5962)
Supplement: Supplementary file 9 — Appendix S9 [file CAM4-12-12504-s005.docx]

Appendix 9. Moderator Analysis for Complete Control of Delayed Vomiting

| **Variables ^a^** | **No. of Studies** |  | **Effect Size and 95% Confidence Intervals** | | |  | **Test of Moderators (*p*)** |
| --- | --- | --- | --- | --- | --- | --- | --- |
|  |  |  | **RR ^b^** | **LL** | **UL** |  |  |
| **Sex** |  |  |  |  |  |  | .504 |
| Female | 1 |  | 1.07 | 0.57 | 2.02 |  | - |
| Mix | 8 |  | 1.56 | 1.02 | 2.40 |  | - |
| **Previous chemotherapy experience** |  |  |  |  |  |  | .681 |
| Mix | 2 |  | 0.92 | 0.22 | 3.92 |  | - |
| No | 1 |  | 1.07 | 0.57 | 2.02 |  | - |
| **Previous acupuncture experience** | 0 |  | - | - | - |  | - |
| **Emetic risk of chemotherapy** |  |  |  |  |  |  | .573 |
| High | 3 |  | 1.22 | 0.57 | 2.61 |  | - |
| Low | 1 |  | 1.38 | 0.93 | 2.04 |  | - |
| Moderate | 2 |  | 1.53 | 0.01 | 186.92 |  | - |
| Moderate or high | 4 |  | 2.18 | 0.76 | 6.22 |  | - |
| **Type of outcome measurement** | 0 |  | - | - | - |  | - |
| **Experience of intervention provider ≥ 5 years** | 0 |  | - | - | - |  | - |
| **Clinical setting** |  |  |  |  |  |  | .407 |
| Inpatient | 6 |  | 1.67 | 1.02 | 2.73 |  | - |
| Outpatient | 1 |  | 1.07 | 0.57 | 2.02 |  | - |
| **Allocation concealment** |  |  |  |  |  |  | - |
| Unclear risk of bias | 10 |  | 1.47 | 1.07 | 2.00 |  | - |
| **Attrition bias** |  |  |  |  |  |  | .345 |
| Low risk of bias | 3 |  | 1.85 | 0.58 | 5.90 |  | - |
| Unclear risk of bias | 7 |  | 1.35 | 0.91 | 2.00 |  | - |
| **Registration of study** | 0 |  | - | - | - |  | - |
| **Clinical design of RCT** |  |  |  |  |  |  | .194 |
| Cross-over | 1 |  | 2.67 | 1.32 | 5.39 |  | - |
| Parallel | 9 |  | 1.37 | 1.01 | 1.86 |  | - |
| **Study center** |  |  |  |  |  |  | .515 |
| Two centers | 1 |  | 1.07 | 0.57 | 2.02 |  | - |
| Single center | 8 |  | 1.46 | 1.05 | 2.01 |  | - |
| **Reported as adequate training of intervention provider ^c^** |  |  |  |  |  |  | .194 |
| Unclear | 9 |  | 1.37 | 1.01 | 1.86 |  | - |
| Judged as adequately trained | 1 |  | 2.67 | 1.32 | 5.39 |  | - |
| **Rescue medication ^c^** |  |  |  |  |  |  | .137 |
| Unclear | 8 |  | 1.42 | 0.94 | 2.15 |  | - |
| Planned to administer additional antiemetics | 2 |  | 1.64 | 0.23 | 11.73 |  | - |

Notes: **a.** Variable categories are listed based on the availability of study-level information (i.e., if the numbers do not add up to the total number of studies, this is due to missing information); **b.** a RR > 1 indicates acupuncture increases the complete control rate, a RR < 1 indicates acupuncture decreases the complete control rate; **c.** Exploratory variable.

Abbreviations: RR, risk ratio; LL, lower limit; UL, upper limit.
