# Supplementary material for: Acupuncture for the prevention of chemotherapy‐induced nausea and vomiting in cancer patients: A systematic review and meta‐analysis
Source: Cancer Med. 2023 May 24;12(11):12504–17. doi: 10.1002/cam4.5962 (PMC10278514; doi:10.1002/cam4.5962)
Supplement: Supplementary file 10 — Appendix S10 [file CAM4-12-12504-s001.pdf]

Risk of Bias Assessment for Included Studies

Risk of bias domains

Study	D1	D2	D3	D4	D5	D6	D7	D8	D9	D10	D11	D12	D13	Overall
Beith JM 2012	+	-	-	-	+	+	?	?	?	?	?	?	-	-
Brinkhaus B 2019	+	+	?	?	?	?	?	?	?	?	?	?	+	+
Cai LH 2011	+	-	X	X	?	?	?	?	?	-	?	-	-	X
Cao HQ 2006	-	-	?	?	?	?	?	?	?	?	?	?	-	-
Chen C 2004	-	-	?	?	?	?	?	?	?	?	?	?	-	-
Chen M 2016	+	-	?	?	?	?	?	?	?	?	?	?	-	-
Chen RB 2011	-	-	X	X	?	?	?	?	?	+	?	+	-	X
Dundee JW 1987	-	-	?	?	?	?	?	?	?	?	?	?	-	-
Guo JY 2016	+	-	X	X	?	?	?	?	?	-	?	-	-	X
Han Y 2018	-	-	?	?	?	?	?	?	?	?	?	?	-	-
Huang KJ 2016	+	-	X	X	?	?	?	?	?	?	?	-	-	X
Huang ZF 2008	+	-	?	?	?	?	?	?	?	?	?	?	-	-
Jiao DP 2008	+	-	?	?	?	?	?	?	?	?	?	?	-	-
Lai HK 2011	-	-	?	?	?	?	?	?	?	?	?	?	-	-
Li D 2017	+	+	X	X	?	?	?	?	-	-	?	?	-	X
Li ML 2016	+	-	?	?	?	?	?	?	?	?	?	?	-	-
Li QL 2007	-	-	?	?	?	?	?	?	?	?	?	?	-	-
Li YH 2014	+	-	?	?	?	?	?	?	?	?	?	?	-	-
Liao GY 2018	+	-	?	?	?	?	?	?	?	?	?	?	-	-
Liu M 2017	+	-	X	X	?	?	?	?	+	+	+	+	-	X
Lv JS 2012	-	+	?	?	?	?	?	?	?	?	?	?	-	-
McKeon C 2015	+	-	?	?	?	?	?	?	?	?	?	?	-	-
Qi J 2018	+	-	?	?	?	?	?	?	?	?	?	?	-	-
Shen BY 2013	+	-	?	?	?	?	?	?	?	?	?	?	-	-
Shen J 2000	+	+	?	?	?	?	?	?	?	?	?	?	-	-
Streitberger K 2003	-	+	-	-	+	+	+	+	?	?	?	?	-	-
Teng HQ 2007	-	-	?	?	?	?	?	?	?	?	?	?	-	-
Wang G 2016	+	-	?	?	?	?	?	?	?	?	?	?	-	-
Wang JC 2019	-	-	?	?	?	?	?	?	?	?	?	?	-	-
Wang YL 2019	+	-	?	?	?	?	?	?	?	?	?	?	-	-
Wu BQ 2011	-	-	X	X	?	?	?	?	?	-	?	-	-	X
Xu Y 2014	-	-	X	X	?	?	?	?	?	-	?	-	-	X
Yan JH 2017	+	-	?	?	?	?	?	?	?	?	?	?	-	-
Zhang J 2017	+	-	X	X	?	?	?	?	+	+	+	+	-	X
Zhang LC 2014	+	-	X	X	?	?	?	?	?	-	?	-	-	X
Zhang X 2014	-	-	X	X	?	-	?	-	?	?	?	?	-	X
Zhou L 2006	-	-	?	?	?	?	?	?	?	?	?	?	-	-
Zhu WJ 2016	+	-	X	X	?	?	?	?	-	-	?	-	-	X

Judgement
- \+ Low
- \- Unclear
- X High
- ? Not applicable

Note

D1: Random sequence generation
D2: Allocation concealment
D3: Performance bias for complete control of nausea and/or vomiting
D4: Detection bias for complete control of nausea and/or vomiting
D5: Attrition bias for complete control of acute nausea (Acupuncture vs Sham)
D6: Attrition bias for complete control of acute vomiting (Acupuncture vs Sham)
D7: Attrition bias for complete control of delayed nausea (Acupuncture vs Sham)
D8: Attrition bias for complete control of delayed vomiting (Acupuncture vs Sham)
D9: Attrition bias for complete control of acute nausea (Acupuncture and usual care vs Usual care)
D10: Attrition bias for complete control of acute vomiting (Acupuncture and usual care vs Usual care)
D11: Attrition bias for complete control of delayed nausea (Acupuncture and usual care vs Usual care)
D12: Attrition bias for complete control of delayed vomiting (Acupuncture and usual care vs Usual care)
D13: Selective outcome reporting
