# Supplementary figures and images for: Acupuncture for the prevention of chemotherapy‐induced nausea and vomiting in cancer patients: A systematic review and meta‐analysis
Source: Cancer Med. 2023 May 24;12(11):12504–17. doi: 10.1002/cam4.5962 (PMC10278514; doi:10.1002/cam4.5962)

## Funnel Plot (Complete Control of Acute Vomiting)

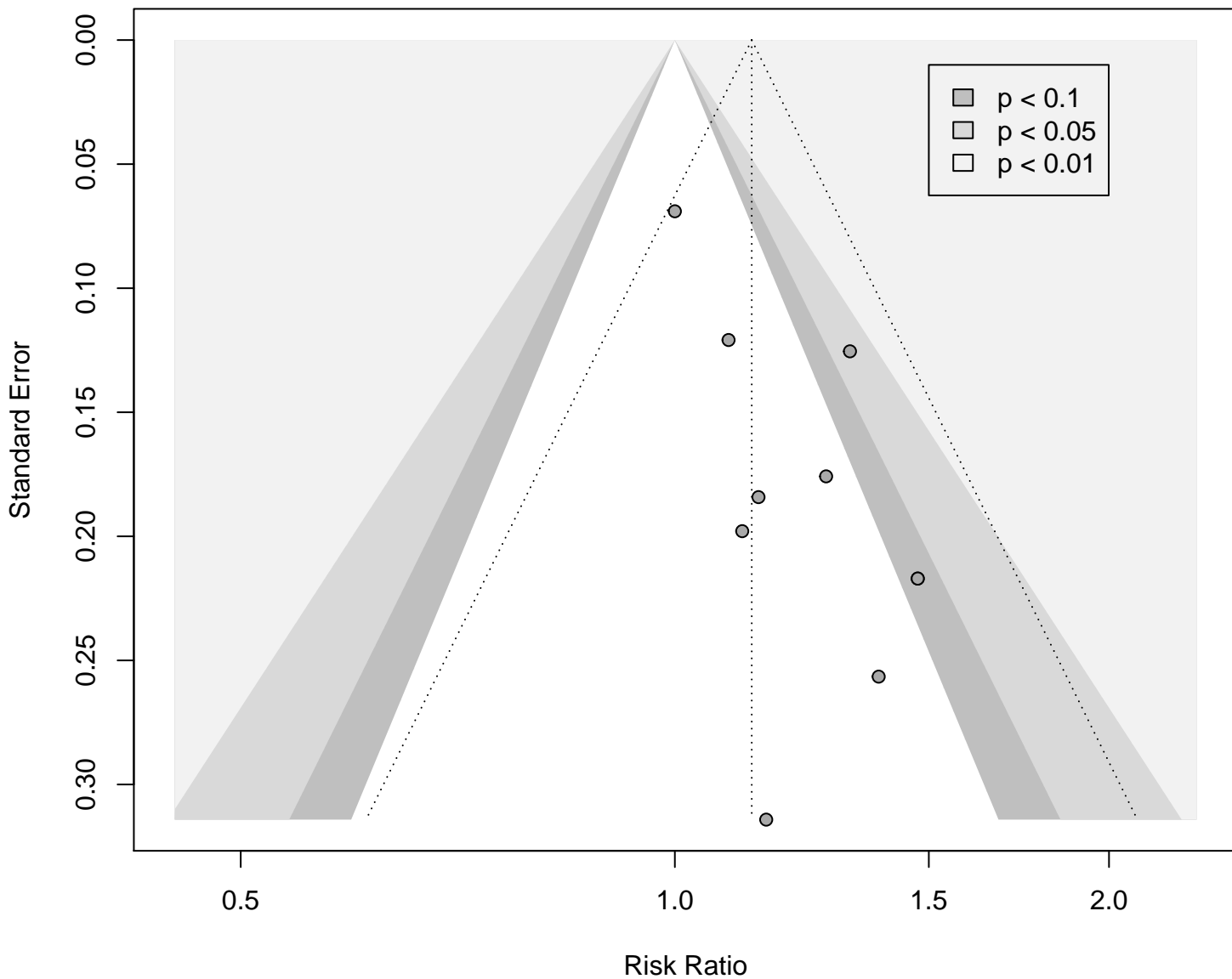

Supplement: Supplementary file 11 — Appendix S11 [file CAM4-12-12504-s010.pdf]

# Funnel Plot (Complete Control of Delayed Vomiting)

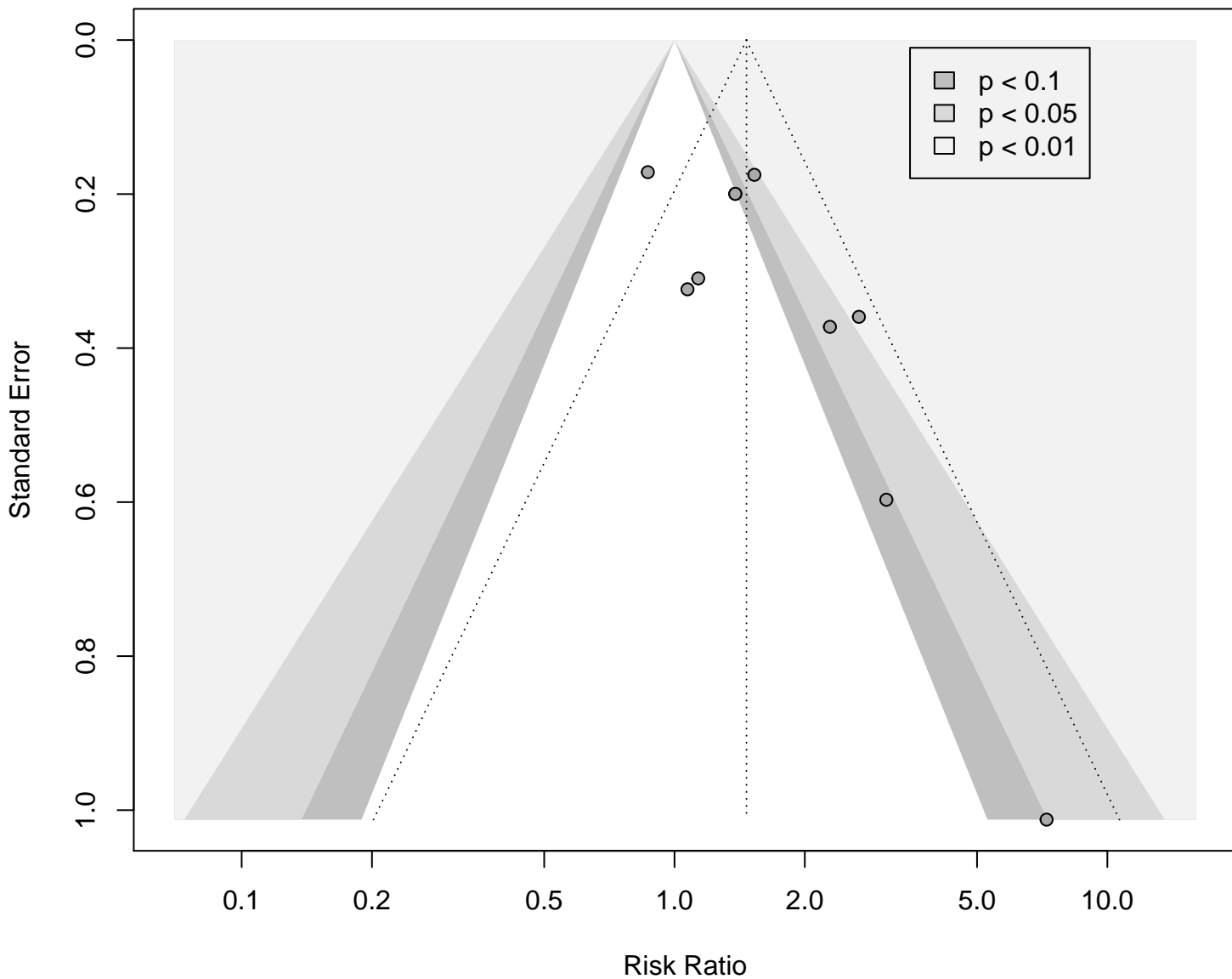

Supplement: Supplementary file 12 — Appendix S12 [file CAM4-12-12504-s006.pdf]
